# The genus *Porana* (Convolvulaceae) - A phytochemical and pharmacological review

**DOI:** 10.3389/fphar.2022.998965

**Published:** 2022-10-18

**Authors:** Yu Peng, Ye Li, Yuanyuan Yang, Yuanqing Gao, Hui Ren, Jing Hu, Xiaomin Cui, Wenjing Lu, Hongxun Tao, Zhiyong Chen

**Affiliations:** ^1^ Shaanxi Academy of Traditional Chinese Medicine, Xi’an, Shaanxi, China; ^2^ Jiangsu Provincial Key Laboratory of Cardiovascular and Cerebrovascular Medicine, School of Pharmacy, Nanjing Medical University, Nanjing, Jiangsu, China; ^3^ Xi’an Institute for Food and Drug Control, Xi’an, Shaanxi, China; ^4^ School of Food and Biological Engineering, Jiangsu University, Zhenjiang, Jiangsu, China

**Keywords:** Porana burm. f., traditional use, phytochemistry, network analysis, pharmacological activity

## Abstract

There are about 20 species of *Porana* Burm. f. worldwide in tropical and subtropical Asia, Africa and neighboring islands, Oceania, and the Americas. In China, India, and other places, this genus enjoys a wealth of experience in folk applications. Nevertheless, the chemical composition of only five species has been reported, and 59 compounds have been isolated and identified, including steroids, coumarins, flavonoids, quinic acid derivatives, and amides. Pharmacological studies revealed that extracts from this genus and their bioactive components exhibit anti-inflammatory, analgesic, antioxidant, anti-gout, anti-cancer, and anti-diabetic effects. Although this genus is abundant, the development of its pharmacological applications remains limited. This review will systematically summarize the traditional and current uses, chemical compositions, and pharmacological activities of various *Porana* species. Network analysis was introduced to compare and confirm its output with current research progress to explore the potential targets and pathways of chemical components in this genus. We hope to increase understanding of this genus’s medicinal value and suggest directions for rational medicinal development.

## 1 Introduction

**FIGURE 1:**
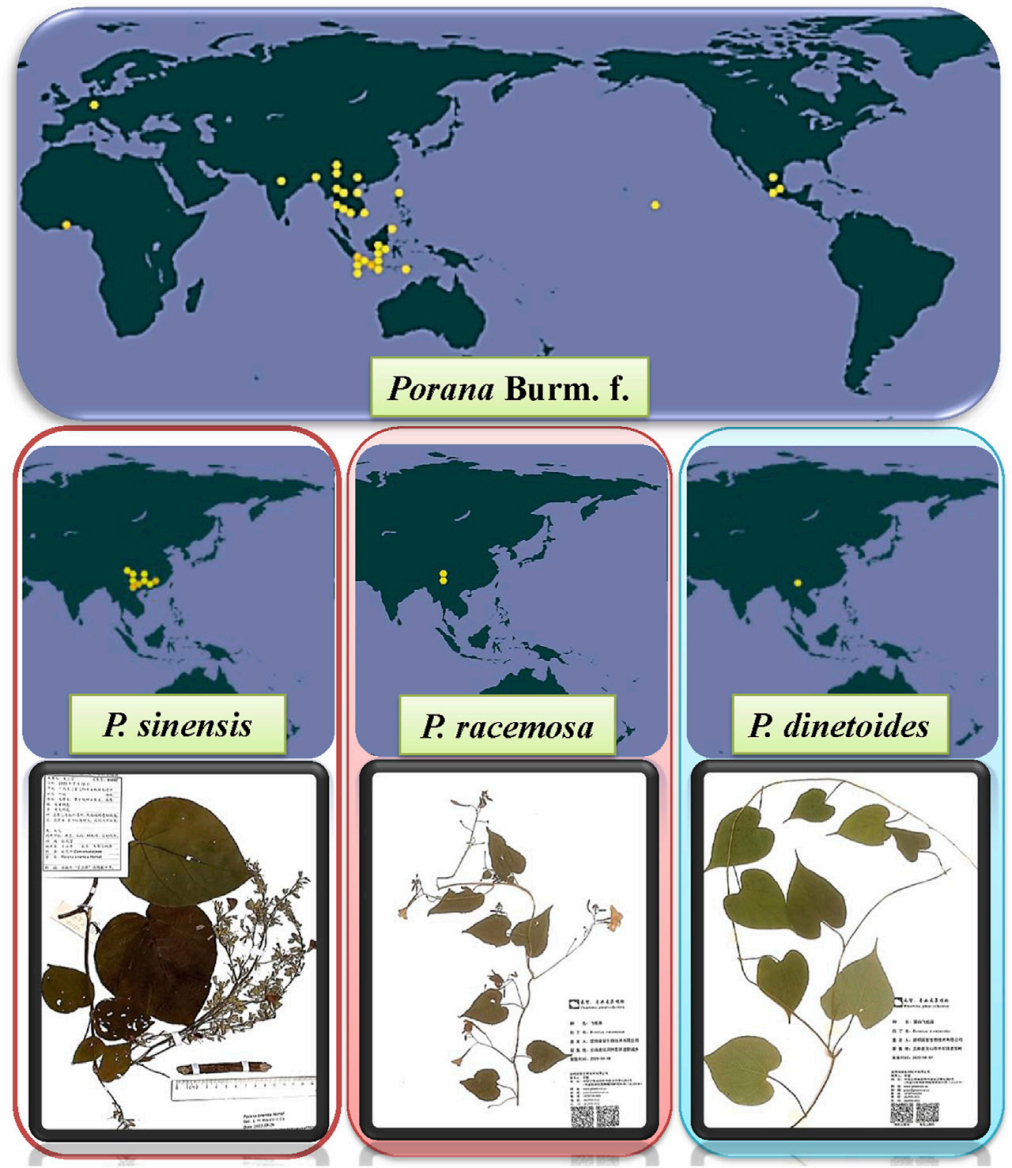
The global distribution of *Porana* Burm. f., and the plant specimens of *P. sinensis*, *P. racemosa*, and *P. dinetoides*.

There are more than 20 species of *Porana* Burm. f. worldwide in tropical and subtropical Asia, Africa and neighboring islands, Oceania, and the Americas. Fifteen species are displayed in Table 1 (for more information, see http://www.plantsoftheworldonline.org or www.theplantlist.org). The global distribution of *Porana* plants based on the Global Biodiversity Information Facility (https://www.gbif.org/) and the herbarium diagrams of three mainstream species are shown in Figure 1.

**TABLE 1:** Synonyms and distribution of *Porana* species.

No.	Species	Synonyms	Distribution
1	*Porana acuminata* P.Beauv	*Neuropeltis acuminata* (P.Beauv.) Benth	West Tropical Africa
2	*Porana densiflora* Hallier f	*Metaporana densiflora* (Hallier f.) N.E.Br	Tanzania
3	*Porana dinetoides* C.K.Schneid	*Dinetus dinetoides* (C.K.Schneid.) Staples	Assam, China South-Central, Myanmar
4	*Porana discifera* C.K.Schneid	*Poranopsis discifera* (C.K.Schneid.) Staples	Assam, China South-Central, Laos, Myanmar, Thailand, Vietnam
5	*Porana duclouxii* Gagnep. & Courchet	*Dinetus duclouxii* (Gagnep. & Courchet) Staples	China South-Central
6	*Porana grandiflora* Wall	*Dinetus grandiflorus* (Wall.) Staples	East Himalaya, Nepal, Tibet
7	*Porana henryi* Verdc	*Poranopsis sinensis* (Hand.-Mazz.) Staples	China South-Central
8	*Porana mairei* Gagnep	*Dinetus decorus* (W.W.Sm.) Staples	Assam, China South-Central, Myanmar
9	*Porana paniculata* Roxb	*Poranopsis paniculata* (Roxb.) Roberty	Assam, Bangladesh, East Himalaya, India, Myanmar, Nepal, Pakistan, Tibet, West Himalaya
10	*Porana parvifolia* (K.Afzel.) Verdc	*Metaporana parvifolia* (K.Afzel.) Verdc	Madagascar
11	*Porana racemosa* Roxb	*Dinetus racemosus* (Roxb.) Sweet	Assam, Bangladesh, China North-Central, China South-Central, China Southeast, East Himalaya, India, Jawa, Laos, Lesser Sunda Is., Myanmar, Nepal, Pakistan, Sulawesi, Thailand, Vietnam, West Himalaya
12	*Porana sinensis* Hemsl	*Tridynamia sinensis* (Hemsl.) Staples	China North-Central, China South-Central, China Southeast, Vietnam
13	*Porana spectabilis* Kurz	*Tridynamia spectabilis* (kurz) Parmar	Andaman Is., Assam, Cambodia, China South-Central, China Southeast, Hainan, Laos, Malaya, Myanmar, Thailand, Vietnam
14	*Porana subrotundifolia* De Wild	*Paralepistemon shirensis* (Oliv.) Lejoly & Lisowski	Angola, KwaZulu-Natal, Malawi, Mozambique, Northern Provinces, Zambia, Zaïre, Zimbabwe
15	*Porana velutina* (M.Martens & Galeotti) Hallier f	*Porana nutans* (Choisy) O'Donell	Mexico Central, Mexico Southwest

*Porana* plants are vines, woody, herbaceous, or climbing shrubs. Their ovate leaves are mostly cordate at the base, with petioles. The inflorescence morphology of *Porana* plants is divided into racemes or panicles, with some single-flower forms. Their bracts are leaflike, small and subulate, or absent. Their corollas are neatly arranged, presenting white, reddish, and some lavender. The ovaries are primarily glabrous. Some are one-celled, containing two ovules, while some are one-to two-celled, containing two to four ovules. Their stigmas are spherical, each connecting to the ovary by one style. Capsules of *Porana* plants are relatively small, sub-globose to oblong, dehiscent in two petals, or not dehiscent. *Porana* plants usually have only one spherical and glabrous seed (Chen et al., 2004).

The medicinal records of *Porana* plants are extensive. *Porana paniculata* Roxb. has been used in folk medicine to treat pain and inflammation in Ayurveda and India (Kumar et al., 2015). *Porana sinensis* Hemsl. is a direct substitute for commercial *Dingongteng* medicinal materials and is known for its therapeutic effect on rheumatoid arthritis and bruises (Ren et al., 2019). According to the National Compendium of Chinese Herbal Medicine, the whole plant of *Porana racemosa* Roxb. is used to treat colds and indigestion (Guoqiang, 2014), while its stems and roots are used to treat rheumatism (Liu and Li, 1997). Research on the phytochemistry of *Porana* plants focuses on *Porana discifera* C.K.Schneid., *P. racemosa*, *P. sinensis*, *Porana spectabilis* Kurz, and *Porana duclouxii* Gagnep. & Courchet; 59 compounds have been isolated from *Porana* plants, including 14 steroids, six coumarins, seven flavonoids, six quinic acid derivatives, and three amides (Zhu et al., 2007; Li et al., 2013; Ding et al., 2014; Chen et al., 2015; Xue et al., 2019). Pharmacological studies revealed that the extracts of *Porana* plants and their bioactive compounds treat arthritis (Dou et al., 2013), gout (Chen et al., 2014; Du et al., 2020), inflammation (Wu et al., 2016; Xue et al., 2019), and cancer (Huang et al., 2019).

Although *Porana* has a wide range of medicinal uses, and its extracts and bioactive compounds show excellent efficacy, current research remains limited, complicating the investigation of its chemical components, pharmacological activities, quality control, and safety. Therefore, it is critical to perform a systematic literature review on *Porana* to promote rational medicinal development.

## 2 Methodology

An extensive search of studies was conducted from scientific journals (original research, reviews, and short communications), books, and reports from internationally recognized databases (Web of Science, PubMed, ScienceDirect, China National Knowledge Infrastructure, and Google Scholar). The following keywords were selected: “*Porana*,” “pharmacology,” “ethnopharmacology,” “compound,” “phytotherapy,” “biological activity,” “substitute,” “toxicity,” and “quality control.” The bibliographies of all selected articles were scanned to seek additional relevant articles.

## 3 Traditional uses

The medicinal parts of *P. sinensis* are canes, which have been used to substitute for the endangered traditional Chinese medicine *Dinggongteng* (*Erycibes caulis*) in China (Xue et al., 2017). *Dinggongteng* is a traditional Chinese folk medicine, first recorded in the Supplement to Medica, which recorded the effect of dispelling wind and strengthening the waist (Shang, 2004). The National Collection of Chinese Herbal Medicine, the Dictionary of Chinese Herbal Medicine, and the Chinese Materia Medica have documented *Dinggongteng*, which dispels wind and dampness, relaxes tendons, activates collaterals, reduces swelling, and relieves pain. The traditional clinical application of *Dinggongteng* has been to treat rheumatoid arthritis, bruises, and other diseases, according to the 2020 edition of the Chinese Pharmacopoeia. With *E. caulis* as the main medicinal material, and more than ten Chinese patent medicines have been developed, including *Feng Liaoxing Rheumatism Dieda Liquor* and *Tengluoning Capsule* (Fan et al., 2021; Peng et al., 2021). *Dinggongteng* is often combined with *Cinnamomi ramulus*, *Ephedrae herba*, *Angelicae sinensis radix*, and other medicinal materials. Wu et al. (2005) investigated the commercial medicinal materials in Guangxi, the main production area for *E. caulis*, as well as Shanghai, Jiangsu, Zhejiang, and other places, and found that the wild resources of *Erycibe obtusifolia* Benth. and *Erycibe schmidtii* Craib could no longer meet the demand for clinical medication. *P. sinensis* has already become a mainstream substitute for *E. caulis* on the market. The widespread application of *P. sinensis* has promoted the sustainable utilization of the endangered traditional Chinese medicine *E. caulis* while accumulating evidence for the effectiveness and safety of *P. sinensis*.

*P. racemosa* is also a traditional folk medicine of the Dai, Yi, and Tujia nationalities in China, and its whole herb is the medicinal part (Fang et al., 2007). According to the National Compendium of Chinese Herbal Medicine, the whole plant of *P. racemosa* relieves the surface, eliminates food accumulation, and is primarily used for colds and food accumulation (Editorial Board, 1975). Its stem and root treat rheumatism (Liu and Li, 1997). In the treatment of cold and fever, it is often used in combination with *Peucedanum praeruptorum* and *Periliae fructus*, while in the treatment of food accumulation, it is often used in combination with *Crataegi fructus* and *Serissa serissoides* (Fang et al., 2007). In Guangxi Province, *P. spectabilis* is used to treat uterine prolapse, with its whole herb as the medicinal part (Li et al., 1985). *P. spectabilis* contains scopoletin, ethyl caffeate, and other compounds (Zhu et al., 2001); however, no pharmacodynamic study has been reported. According to the Chinese Materia Medica, the root of *Porana mairei* Gagnep. relieves cough and asthma (Editorial Board, 2009).

In summary, *Porana* plants are used as folk medicines. The genus has received increasing attention due to the widespread use of *P. sinensis* as a substitute for *E. caulis*.

## 4 Chemical compositions of *Porana* plants

**FIGURE 2:**
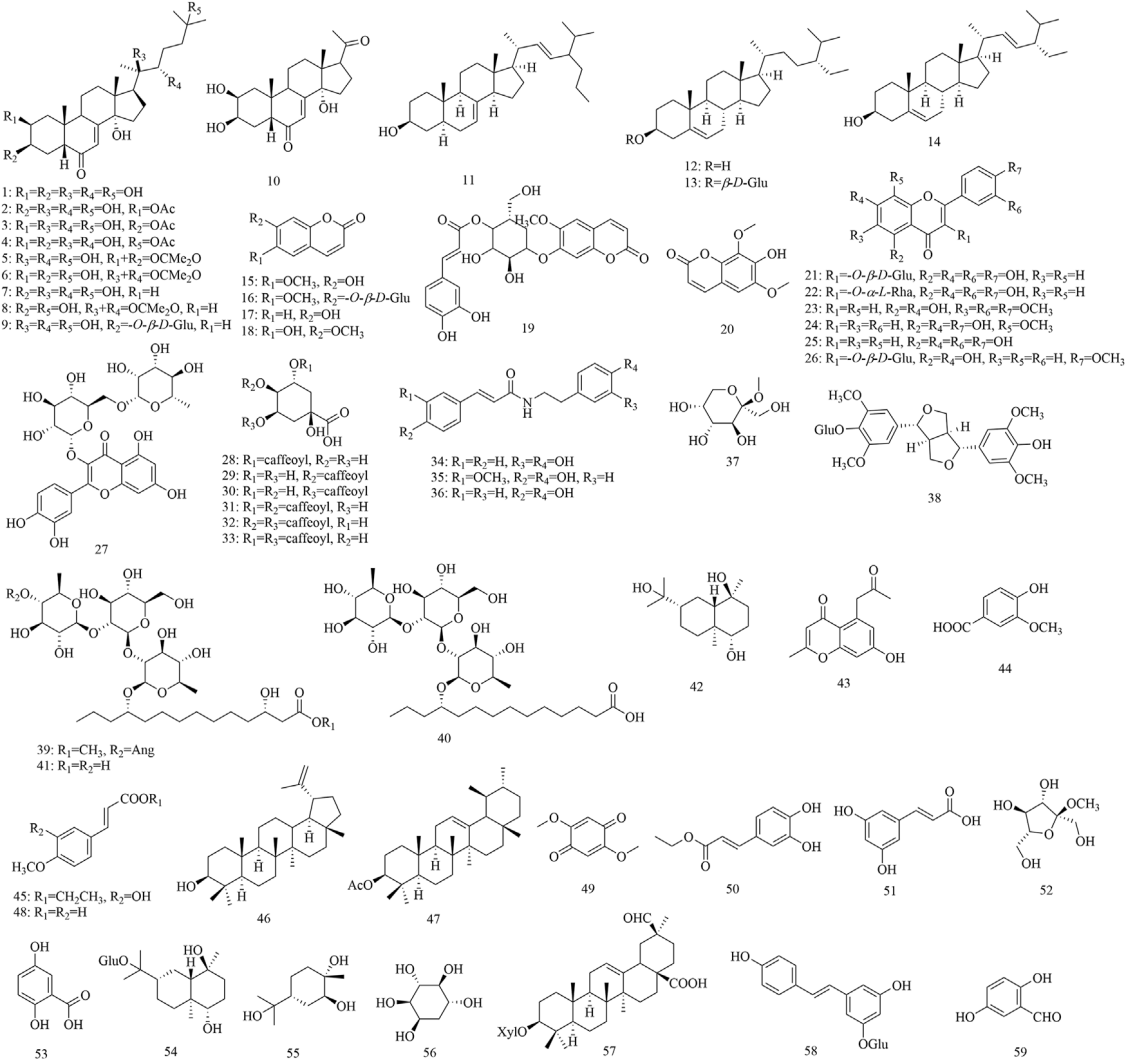
Structural formulas of chemical components of genus *Porana*.

Based on literature reports and our previous research, we concluded that the research on the phytochemical constituents of this genus focused on *P. discifera*, *P. racemosa*, *P. sinensis*, *P. spectabilis*, and *P. duclouxii*. Fifty-nine compounds have been isolated from *Porana* species, including 14 steroids, six coumarins, seven flavonoids, six quinic acid derivatives, three amides, and 23 other compounds. These compounds are displayed in Table 2 according to their chemical name, chemical type, and their original plants. The structural formulas of these compounds are shown in Figure 2.

**TABLE 2:** Chemical compositions of *Porana* plants.

No	Compounds	Molecular formula	Type	Plant parts and species	References
1	β-ecdysterone	C_27_H_44_O_7_	Steroids	Aerial parts of *P. discifera*	Zhu et al. (2000)
2	β-ecdysterone-2-acetate	C_29_H_46_O_8_	Steroids	Aerial parts of *P. discifera*	Zhu et al. (2000)
3	β-ecdysterone-3-acetate	C_29_H_46_O_8_	Steroids	Aerial parts of *P. discifera*	Zhu et al. (2000)
4	β-ecdysterone-25-acetate	C_29_H_46_O_8_	Steroids	Aerial parts of *P. discifera*	Zhu et al. (2000)
5	2,3-acetonide-β-ecdysterone	C_30_H_48_O_7_	Steroids	Aerial parts of *P. discifera*	Zhu et al. (2000)
6	20,22-acetonide-β-ecdysterone	C_30_H_48_O_7_	Steroids	Aerial parts of *P. discifera*	Zhu et al. (2000)
7	2-deoxy-20-hydroxyecdysone	C_27_H_44_O_6_	Steroids	Aerial parts of *P. discifera*	Zhu et al. (2000)
8	2-deoxyecdysterone-20,22-acetonide	C_30_H_48_O_6_	Steroids	Aerial parts of *P. discifera*	Zhu et al. (2000)
9	2-deoxyecdysterone-3-O-β-D-glucopyranoside	C_33_H_54_O_11_	Steroids	Aerial parts of *P. discifera*	Zhu et al. (2000)
10	Posterone	C_21_H_30_O_5_	Steroids	Aerial parts of *P. discifera*	Zhu et al. (2000)
11	Racemosol	C_30_H_50_O	Steroids	Whole plants of *P. racemosa*	Li et al. (2004)
12	β-sitosterol	C_29_H_50_O	Steroids	Stems and roots of *P. racemosa*	Liu and Li, (1997); Yu et al., (2003); Zhang et al., (2006)
				Stems of *P. sinensis*	
				Leaves and stems of *P. discifera*	
13	β-daucosterol	C_35_H_60_O_6_	Steroids	Whole plants of *P. racemosa*	Wang, (2003); Yu et al., (2003); Zhang et al., (2006)
				Stems of *P. sinensis*	
				Leaves and stems of *P. discifera*	
14	Stigmasterol	C_29_H_48_O	Steroids	Whole plants of *P. racemosa*	Wang, (2003)
15	Scopoletin	C_10_H_8_O_4_	Coumarins	Stems of *P. sinensis*	Zhu, (2001); Yu et al., (2003); Li et al., (2004); Xue et al., (2019)
				Whole plants of *P. racemosa*	
				Leaves and stems of *P. discifera*	
				Barks of *P. spectabilis*	
16	Scopolin	C_16_H_18_O_9_	Coumarins	Stems of *P. sinensis*	Zhu, (2001); Yu et al., (2003); Li et al., (2004); Xue et al., (2019)
				Whole plants of *P. racemosa*	
				Leaves and stems of *P. discifera*	
				Barks of *P. spectabilis*	
17	Umbelliferone	C_9_H_6_O_3_	Coumarins	Whole plants *P. racemosa*	Li et al. (2004)
18	Isoscopoletin	C_10_H_8_O_4_	Coumarins	Stems of *P. sinensis*	Xue et al. (2019)
19	7-*O*-[4′-*O*-(3″,4″-dihydroxycinnamyl)-*β*-D-glucopyranosyl]-6-methoxycoumarin	C_26_H_26_O_11_	Coumarins	Stems of *P. sinensis*	Xue et al. (2019)
20	Isofraxidin	C_11_H_10_O_5_	Coumarins	Leaves and stems of *P. discifera*	Yu et al. (2003)
21	Quercetin-3-*O-β*-D-glucopyranoside	C_21_H_20_O_12_	Flavonoids	Whole plants of *P. racemosa*	Li et al. (2004)
22	Quercetin-3-*O-α*-L-rhamnopyranoside	C_21_H_20_O_11_	Flavonoids	Whole plants of *P. racemosa*	Li et al. (2004)
23	Eupatilin	C_18_H_16_O_7_	Flavonoids	Whole plants of *P. racemosa*	Li et al. (2004)
24	4ʹ-Hydroxywogonin	C_16_H_12_O_6_	Flavonoids	Leaves and stems of *P. discifera*	Yu et al. (2003)
25	Quercetin	C_15_H_10_O_7_	Flavonoids	Leaves and stems of *P. discifera*	Wang, (2003); Yu et al., (2003)
				Whole plants of *P. racemosa*	
26	Kaempferol-3-*O-β*-D-glucopyranoside	C_21_H_20_O_11_	Flavonoids	Whole plants of *P. racemosa*	Wang, (2003)
27	Rutin	C_27_H_30_O_16_	Flavonoids	Whole plants of *P. racemosa*	Wang, (2003)
28	Chlorogenic acid	C_16_H_18_O_9_	Quinic acid derivatives	Stems of *P. sinensis*	Chen et al., (2013); Chen et al., (2019); Chen et al., (2020)
29	4-*O*-caffeoylquinic acid	C_16_H_18_O_9_	Quinic acid derivatives	Stems of *P. sinensis*	Chen et al., (2019); Chen et al., (2020)
30	5-*O*-caffeoylquinic acid	C_16_H_18_O_9_	Quinic acid derivatives	Stems of *P. sinensis*	Chen et al., (2019); Chen et al., (2020)
31	3,4-dicaffeoylquinic acid	C_25_H_24_O_12_	Quinic acid derivatives	Stems of *P. sinensis*	Chen et al., (2019); Chen et al., (2020)
32	4,5-dicaffeoylquinic acid	C_25_H_24_O_12_	Quinic acid derivatives	Stems of *P. sinensis*	Chen et al., (2019); Chen et al., (2020)
33	3,5-dicaffeoylquinic acid	C_25_H_24_O_12_	Quinic acid derivatives	Stems of *P. sinensis*	Chen et al., (2019); Chen et al., (2020)
34	(*E*)-*N*-2-(2,3-dihydroxyphenyl) ethyl cinnamamide	C_17_H_17_NO_3_	Amides	Whole plants of *P. racemosa*	Li et al. (2004)
35	*N-trans*-feruloyltyramine	C_18_H_19_NO_4_	Amides	Stems of *P. sinensis*	Zhang et al. (2006)
36	*N-trans*-coumaroyltyramine	C_17_H_17_NO_3_	Amides	Stems of *P. sinensis*	Zhang et al. (2006)
37	Methyl *β-D*-frucopyranoside	C_7_H_14_O_6_	Others	Whole plants of *P. racemosa*	Zhu et al., (2001); Li et al., (2004)
				*Barks of P. spectabilis*	
38	Syringaresinol-4-*O-β-D*-glucopyranoside	C_28_H_36_O_13_	Others	Whole plants of *P. racemosa*	Zhu et al., (2001); Li et al., (2004)
				*Barks of P. spectabilis*	
39	Poranaside A	C_38_H_66_O_18_	Others	Roots of *P. duclouxii*	Ding et al. (2014)
40	Poranic acid A	C_32_H_58_O_16_	Others	Roots of *P. duclouxii*	Ding et al. (2014)
41	Poranic acid B	C_32_H_58_O_17_	Others	Roots of *P. duclouxii*	Ding et al. (2014)
42	Disciferitriol	C_15_H_28_O_3_	Others	Aerial parts of *P. discifera*	Zhu et al. (2007)
43	Cassiachromone	C_13_H_12_O_4_	Others	Leaves and stems of *P. discifera*	Yu et al. (2003)
44	Vanillic acid	C_8_H_8_O_4_	Others	Whole plants of *P. racemosa*	Wang, (2003)
45	Ethyl 4′-hydroxy-3′-methoxycinnamate	C_12_H_14_O_4_	Others	Whole plants of *P. racemosa*	Wang, (2003)
46	Lupeol	C_30_H_50_O	Others	Whole plants of *P. racemosa*	Wang, (2003)
47	α-amyrin acetate	C_32_H_52_O_2_	Others	Whole plants of *P. racemosa*	Wang, (2003)
48	4-methoxycinnamic acid	C_10_H_10_O_3_	Others	Whole plants of *P. racemosa*	Wang, (2003)
49	2,5-dimethoxy-1,4-benzoquinone	C_8_H_8_O_4_	Others	Stems of *P. sinensis*	Zhang et al. (2006)
50	Ethyl caffeate	C_11_H_12_O_4_	Others	Stems of *P. sinensis*	Zhu et al., (2001); Zhang et al., (2006)
				Barks of *P. spectabilis*	
51	3-(3,5-dihydroxyphenyl)-2*E*-propenoic acid	C_9_H_8_O_4_	Others	Barks of *P. spectabilis*	Zhu et al. (2001)
52	Methyl *α-D*-frucofuranoside	C_7_H_14_O_6_	Others	Barks of *P. spectabilis*	Zhu et al. (2001)
53	2,5-dihydroxybenzoic acid	C_7_H_6_O_4_	Others	Barks of *P. spectabilis*	Zhu et al. (2001)
54	Disciferoside A	C_21_H_38_O_8_	Others	Aerial parts of *P. discifera*	Zhu, (2001)
55	(4R)-menthane-1S,2S,8-triol	C_10_H_20_O_3_	Others	Aerial parts of *P. discifera*	Zhu, (2001)
56	1β,2β,3α,4β,5α-cyclohexanepentol	C_6_H_12_O_5_	Others	Aerial parts of *P. discifera*	Zhu, (2001)
57	Dodecandral-3-*O-β*-D-xylopyranoside	C_38_H_54_O_4_	Others	Aerial parts of *P. discifera*	Zhu, (2001)
58	*E*-piceid	C_20_H_22_O_8_	Others	Aerial parts of *P. discifera*	Zhu, (2001)
59	2,5-dihydroxybenzaldehyde	C_7_H_6_O_3_	Others	Aerial parts of *P. discifera*	Zhu, (2001)

### 4.1 Steroids

Fourteen steroids have been isolated from *Porana* species, of which 12 were isolated from *P. discifera*, including compounds **1–10** (Zhu et al., 2000) and **12–13** (Yu et al., 2003); four were found in *P. racemosa*, including compounds **11–14** (Liu and Li, 1997; Wang, 2003; Li et al., 2004); two were found in *P. sinensis*, including compounds **12–13** (Zhang et al., 2006). Compounds **1–10** are phytoecdysteroids, natural polyhydroxylated compounds with a four-ringed skeleton, usually comprising 27 carbon atoms or 28–29 carbon atoms with the characteristic 7-en-6 ketone on the steroid nucleus (Tarkowská and Strnad, 2016). Phytoecdysteroids are a class of natural steroids with insect ecdysis activity. They also exhibit extensive pharmacological effects on higher animals, including hypoglycemia, wound repair, and immune regulation (Taha-Salaime et al., 2019; Yusupova et al., 2019). Compounds **1–7** have no anti-inflammatory, sedative, anti-convulsant, or anti-cerebra-hypoxic activities in animal testing with Kunming mice (Zhu et al., 2000). Most steroids reported in *Porana* species have been found in *P. discifera*. In this case, several issues need to be addressed. Are these compounds also present in other plants of this genus, and can they be used as the chemical indicators of the *Porana* Burm. f.? Answering these questions must address the biological activity of steroids among the pharmacological activities of this genus.

### 4.2 Coumarins

Three coumarin compounds have been isolated from *P. racemosa*, including compounds **15–17** (Li et al., 2004). Four coumarin compounds have been found in *P. sinensis*, including compounds **15–16** (Zhang et al., 2006) and **18–19** (Xue et al., 2019). Three coumarin compounds have been reported in *P. discifera*, including compounds **15–16 and 20** (Yu et al., 2003). Two coumarin compounds are found in *P. spectabilis*, including compounds **15–16** (Zhu et al., 2001). The coumarins obtained from *Porana* plants are simple coumarins, and compounds **15** and **16** have been found in four species; these are thought to be the primary pharmacodynamic substances and chemical indicators of *E. caulis* (Chen et al., 2014; Chen et al., 2020). Therefore, compounds **15** and **16** are essential for applying *P. sinensis* as a substitute for *E. caulis.*

### 4.3 Flavonoids

Six flavonoids have been isolated from *P. racemosa*, including compounds **21–23** (Li et al., 2004) and **25–27** (Wang, 2003). Two flavonoids were found in *P. discifera*, including compounds **24–25** (Yu et al., 2003). Flavonoids are very common in plants. According to reports, no characteristic flavonoid has been found in this genus; this might be due to the lack of reports on the chemical constituents of *Porana* plants. However, several characteristic isoflavones, pterocarpans, and rotenoids were identified in *Erycibes* plants (Peng et al., 2021). Based on this, we speculate that flavonoids might be the components differentiating *Porana* from *Erycibes*. Considering flavonoids’ excellent biological activity, exploring such compounds should not be ignored.

### 4.4 Quinic acid derivatives

**FIGURE 3:**
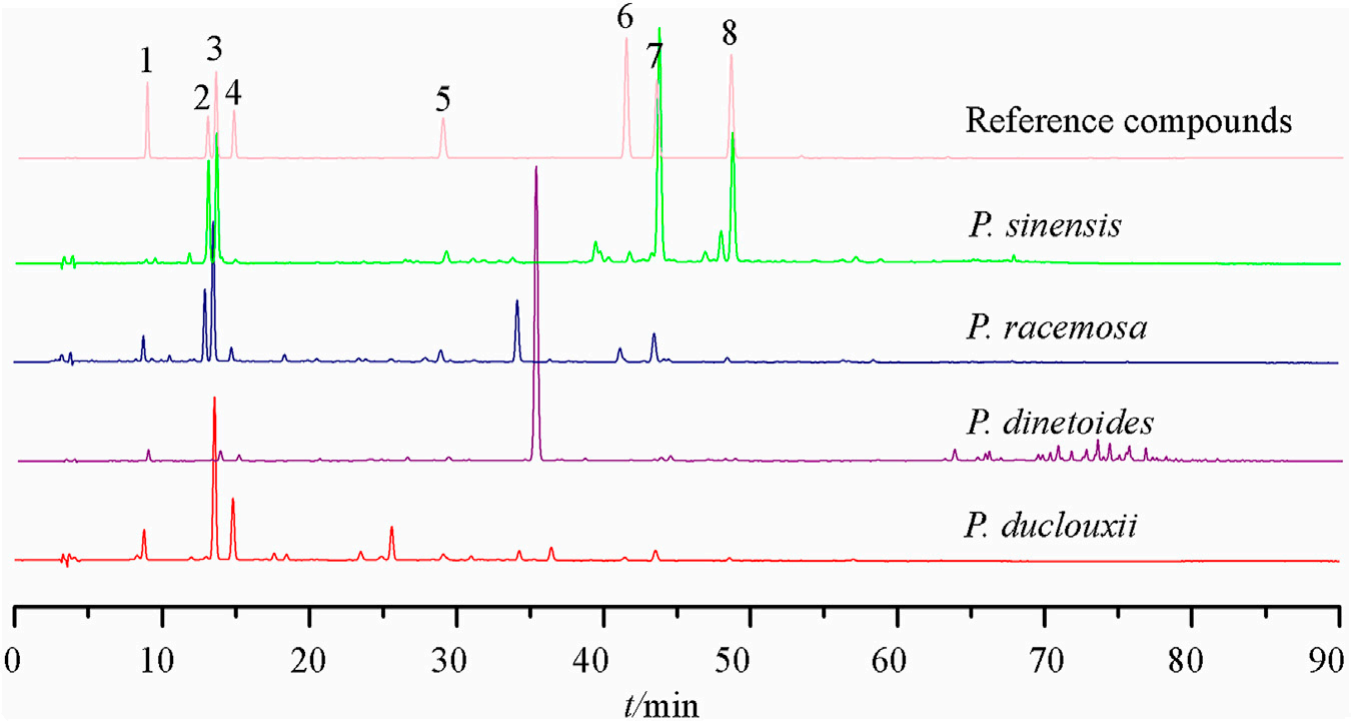
HPLC fingerprints of four species of genus *Porana* 1) 5-*O*-caffeoylquinic acid; 2) Scopolin; 3) Chlorogenic acid; 4) 4-*O*-caffeoylquinic acid; 5) Scopoletin; 6) 3,5-dicaffeoylquinic acid; 7) 3,4-dicaffeoylquinic acid; 8) 4,5-dicaffeoylquinic acid.

Six quinic acid derivatives have been reported in the *Porana* species, including compounds **28–33** (Chen et al., 2013; Chen et al., 2019; Chen et al., 2020), all from *P. sinensis*. Our fingerprint study has revealed that *Porana dinetoides* C.K.Schneid*.*, *P. racemosa*, and *P. duclouxii* also contained quinic acid derivatives (Figure 3). Because many quinic acid derivatives have been detected in fingerprints, this group of compounds can be used as chemical markers for quality control, and this potential deserves further evaluation.

### 4.5 Amides

Three amides have been isolated from *Porana* plants, among which compound **34** has been found in *P. racemosa* (Li et al., 2004) and compounds **35–36** have been found in *P. sinensis* (Zhang et al., 2006). The chemical structures of the three amides are similar. It was reported that compound **36** has better activity than compound **35** in inhibiting nitric oxide (NO) release from lipopolysaccharide (LPS)-induced RAW 264.7 cells, suggesting that introducing a methoxy group at the two-position reduces the anti-inflammatory activity of these compounds (Zheng et al., 2018).

### 4.6 Other compounds

Twenty-three compounds were found in *Porana* species, including one lignin (compound **38**), one monoterpenoid (compound **55**), two sesquiterpenes (compound **42, 54**), three triterpenoids (compound **46, 47, 57**), one benzoquinone (compound **49**), seven phenols (compounds **44, 45, 48, 50, 51, 53, 59**), one stilbene (compound **58**), five glycosides compounds (compound **37, 39–41, 52**), one chromone (compound **43**), and one cyclohexanol (compound **56**). There are many phenolic acids and their derivatives in *Porana* plants. Resin glycosides are characteristic of constituents in Convolvulaceae, and three such components (compounds **39–41**) have been isolated from *Porana* plants (Ding et al., 2014). Compounds **39–41** all have a common trisaccharide moiety and (11*S*)-hydroxytetradecanoic acid or (3*S*,11*S*)-dihydroxytetradecanoic acid as the aglycone. These 23 compounds have not shown any regularity. There is no evidence to assess the importance of these compounds regarding quality control or biological activity.

In summary, only five species of *Porana* plants have been reported, with a total of 59 compounds to date. Combined with the literature reports and fingerprints, phenolic acids and coumarins are widely represented in this genus. Phytoecdysteroids and resin glycosides have specific characteristics; however, their distribution is narrow in this genus. This finding suggests that there might be substantial differences in the chemical compositions of these plants, and a phytochemical study of other species needs to be performed urgently.

## 5 Pharmacological activities of *Porana* plants

### 5.1 Network analysis of *Porana* plants

Because the research on this genus is not systematic, to maximize its medicinal value, we first predicted its targets based on its chemical components using network analysis. Using follow-up comparisons with reported pharmacological research results, the pharmacological effects of this genus were explored.

#### 5.1.1 Enrichment of critical targets

The two-dimensional structures of all 59 compounds found in *Porana* plants were identified in the PubChem database (https://pubchem.ncbi.nlm.nih.gov/search/), their sdf files were downloaded, and they were imported into the Swiss Target Prediction database (http://www.swisstargetprediction.ch/) to predict their targets (Gfeller et al., 2014). After removing the duplicate targets, the potential targets were obtained. We obtained 713 targets in this manner.

#### 5.1.2 The construction and topological parameter analysis of a protein-protein interaction network

All 713 targets obtained in section 5.1.1 were imported into the STRING platform (https://string-db.org/) to construct a PPI network. The topological parameters of the PPI network were calculated and analyzed using Cytoscape 3.6.0. The critical targets were determined with greater values of the degree, closeness centrality, and betweenness centrality than the mean value. This analysis revealed that the mean degree of potential target nodes was 39.5, the mean value of closeness centrality was 0.4326, and the mean value of betweenness centrality was 0.0019. The output was 135 targets with a higher value than the corresponding mean.

#### 5.1.3 Kyoto encyclopedia of genes and genomes pathway enrichment analysis

**FIGURE 4:**
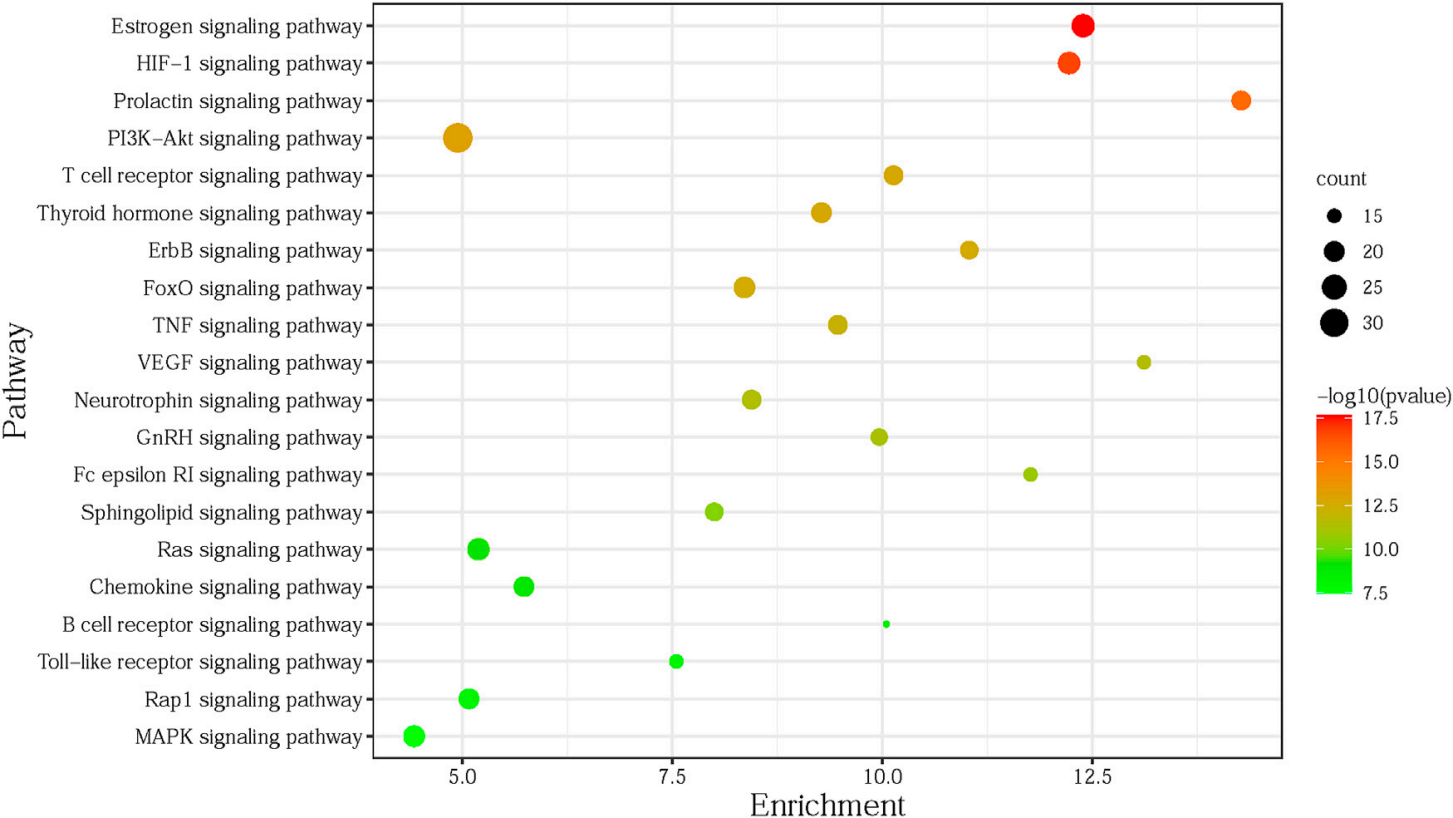
Analysis of KEGG pathway.

To explore the related signaling pathways of the 135 targets obtained in Section 5.1.2, the targets were imported into DAVID (https://david.ncifcrf.gov/home.jsp), with the species limited to humans. KEGG pathway enrichment analysis was performed to identify the relevant signaling pathways. After removing specific diseases such as prostate cancer, viral carcinogenesis, glioma, or other irrelevant items, with *p* < 0.01 as the screening condition, the top 20 most significant pathways were selected for the subsequent enrichment analysis using R language software (Supplementary Table S1). As shown in Figure 4, the abscissa (enrichment) of the bubble chart represents the ratio of the core targets involved in each pathway to the total number of targets in the pathway; the size of the bubble represents the number of core targets involved in the pathway; the color ranges from red to green, indicating that the *p*-value is from small to large, and deeper redness indicates the higher significance of the pathway.

#### 5.1.4 The construction and analysis of the compound-target-pathway network

According to the top 20 pathways of gene enrichment in the KEGG pathway enrichment analysis, the potential targets and the corresponding components enriched in these pathways were outputted. The data table of the C-T-P was imported into Cytoscape 3.6.0 to construct the C-T-P network with a total of 148 nodes (20 pathways, 73 targets, 55 components) and 772 edges. Then the Network Analyzer was used to calculate the topology parameters of the C-T-P network, while a Degree Sorted Circle Layout was applied to lay out nodes. The C-T-P network topology parameters were also analyzed using Network Analyzer, and the results are displayed in Supplementary Table S2. The mean degree of the 55 differentially active components was 7.29, the mean value of closeness centrality was 0.3505, and the mean value of betweenness centrality was 0.0067. Three network topology parameters with 17 components were higher than the corresponding mean value (compounds **1–5, 7, 16–17, 23–25, 34–36, 45, 48,** and **50**). The mean degree of the 73 potential target nodes was 10.58, the mean value of closeness centrality was 0.3732, and the mean value of betweenness centrality was 0.0122. Three network topology parameters of 20 targets were higher than the corresponding mean value (MAPK1, PIK3CA, AKT1, MAP2K1, MAPK3, EGFR, MMP2, PRKCA, ESR2, GSK3B, MAPK14, ESR1, PIK3R1, NRAS, SRC, PTGS2, MMP9, TNF, KDR, and ADORA3). The mean degree of the 20 pathways was 18.55, the mean value of closeness centrality was 0.4054, and the mean betweenness centrality was 0.0254. Three network topology parameters of six signaling pathways were higher than the corresponding mean value (PI3K-Akt, HIF-1, estrogen, MAPK, chemokine, and the thyroid hormone signaling pathway).

The results of the network analysis revealed 17 active compounds in *Porana* species, including six steroids, three flavonoids, three amides, two coumarins, and three organic acid esters. In the follow-up quality control study, critical research should be carried out on the actual content of these compounds. Coumarins are widely distributed in *Porana* species, presenting in *P. sinensis*, *P. racemosa*, *P. discifera*, and *P. spectabilis*. Taking coumarin scopolin as an example, its targets include GSK3B, EGFR, MAPK1, IL2, HSPA8, MMP9, HK1, GAPDH, TNF, ADORA3, acting on PI3K-Akt, HIF-1, estrogen, MAPK, and other signaling pathways. Scopolin promotes the differentiation of osteoblasts and inhibits the decrease of bone mineral density, participating in osteoporosis treatment (Park et al., 2020), possibly associated with the regulation of the estrogen pathway. Intraperitoneal injection of scopolin alleviates the symptoms of adjuvant arthritis in rats by inhibiting inflammatory responses and angiogenesis (Pan et al., 2009); the mechanisms might involve the PI3K-Akt, HIF-1, and MAPK signaling pathways (Park et al., 2015; Qu et al., 2016; Yang et al., 2018).

*Porana* plants are widely used in traditional Chinese and Indian medicine to relieve inflammation and pain and to treat rheumatoid arthritis. Recent studies demonstrated that the PI3K-Akt pathway inhibits apoptosis in chondrocytes, and modulation of the pathway might be a potential target for the therapy of rheumatic arthritis (Malemud, 2015). HIF-1α increases the production of inflammatory cytokines and promotes angiogenesis in rheumatic arthritis patients (Park et al., 2015). We reported that the 40% ethanol extract of *P. sinensis* alleviates rheumatoid arthritis by regulating the PI3K-Akt and HIF-1 signaling pathways (Hu et al., 2022).

*P. racemosa* is another plant in the genus *Porana* with well-documented medicinal applications, which could be used for the treatment of colds. The results of network analysis revealed its primary active components to be scopolin, umbelliferone, eupatilin, and quercetin, which act on AKT1, EGFR, MAPK1, NFκB1, PIK3R1, SRC, TNF, and other targets to regulate PI3K-Akt, MAPK, and the chemokine signaling pathway, indicating the main involvement of inflammatory pathway.

MAPK participates in cell proliferation, differentiation, transformation, and apoptosis regulation through phosphorylation of nuclear transcription factors, cytoskeletal proteins, and enzymes (Yeung et al., 2018). PI3K-Akt regulates survival, cell growth, differentiation, cellular metabolism, and cytoskeletal reorganization of cells. Modification of this pathway is strongly implicated in the pathogenesis of most cancers (Malemud, 2015). The treatment of cancers is not a traditional application of *Porana* plants. Due to the regulatory effect of compounds on multiple anti-cancer pathways, the genus *Porana* has excellent application prospects in anti-cancer drugs.

The targeting pathway of the chemical constituents of *Porana* species supports the application of this genus in the treatment of rheumatoid arthritis, colds, and cancer. However, the application of *Porana* plants in treating these diseases needs to be verified in animal and clinical trials.

### 5.2 Pharmacological activities of the extracts of *Porana* plants

For the extracts, various preparation methods lead to significant differences in chemical composition and bioactivities. When reviewing the pharmacological effects of *Porana* extracts, we focused on the following to facilitate identifying the reasons for the differences in pharmacological effects: plant origin and part, extraction methods, quality control methods, biological activities, and screening models (Table 3). Because *in vitro* studies of extracts have not considered systemic absorption or metabolism of active compounds, the results of these studies might be biased.

**TABLE 3:** Bioactivities of the extracts of *Porana* plants.

No.	Extracts	Species	Part	Condition	Quality control	Activity	Model	Results	References
1	40% ethanol extract	*P. sinensis*	Stem	40% ethanol ultrasonic extraction	HPLC, scopolin 20.07 mg/g, chlorogenic acid 33.86 mg/g, scopoletin 7.68 mg/g plant material	Anti-inflammatory and anti-nociceptive activities	*In vivo*: Xylene-induced ear edema, formalin induced inflammation, carrageenan-induced mice air pouch inflammation in mice, acetic acid-induced writhing, formalin-induced nociception; ig, 143, 285, and 570 mg/kg; positive control: dexamethasone 2 mg/kg, aspirin 200 mg/kg, paracetamol 100 mg/kg	Inhibit the ear swelling, the synthesis of PGE2, reduce the number of writings, and relieve phase II pain in mice	Chen et al. (2013)
2	80% methanol extract	*P. sinensis*	Stem	80% methanol ultrasonic extraction	HPLC, scopolin 1.95 mg/g, chlorogenic acid 2.55 mg/g, scopoletin 0.25 mg/g plant material	Anti-inflammatory activity	*In vitro*: LPS-induced RAW 264.7 cells; 25, 50, 100 μg/ml	Inhibit LPS-induced RAW 264.7 release of NO, and iNOS, COX-2 and IL-6 mRNA expression	Xue et al. (2017)
3	40% ethanol extract	*P. sinensis*	Stem	40% ethanol reflux extraction	HPLC, 5-*O*-caffeoylquinic acid 13.4268 mg/g, scopolin 12.6935 mg/g, chlorogenic acid 48.5457 mg/g, 4-*O*-caffeoylquinic acid 8.2953 mg/g, scopoletin 20.9330 mg/g, 3,4-dicaffeoylquinic acid 28.6063 mg/g, 3,5-dicaffeoylquinic acid 13.5660 mg/g, 4,5-dicaffeoylquinic acid 18.3498 mg/g plant material	Anti-inflammatory activity	*In vitro*: LPS-induced RAW 264.7 cells; 120 μg/ml; positive control: methotrexate 120 μg/ml	Inhibit the release of NO, TNF-α, IL-1β and IL-6 in LPS-induced RAW 264.7 cell; attenuate the severity, pathological changes, and release of cytokines (IL-6 and HIF-1α) during rheumatoid arthritis progression by regulating the PI3K/AKT and HIF-1 pathways	Hu et al. (2022)
							*In vivo*: Collagen-induced arthritis model; ig, 0.6, 0.3, and 0.15 g/kg; positive control: methotrexate 1 mg/kg		
4	60% ethanol extract	*P. paniculata*	Whole plants	Cold maceration method	Total flavonoids 59.86 mg/g of quercetin, total phenols 33.34 mg/g of gallic acid	Anti-oxidant Activity	*In vitro*: DPPH assay, superoxide anion scavenging activity assay, nitric oxide scavenging activity assay, hydrogen peroxide scavenging assay and metal chelating activity; 20, 40, 60, 80 and 100 μg/ml; positive control: L-ascorbic acid, butylated hydroxyanisole, alpha tocopherol, 20, 40, 60, 80 and 100 μg/ml	Present good anti-oxidant activity	Kumar et al. (2015)
5	80% methanol extract of ten samples	*P. sinensis*	Stem	80% methanol ultrasonic extraction	HPLC, chlorogenic acid, 4-*O*-caffeoylquinic acid, 5-*O*-caffeoylquinic acid, 3,4-dicaffeoylquinic acid, 4,5-dicaffeoylquinic acid, 3,5-dicaffeoylquinic acid, scopolin, scopoletin	Anti-oxidant Activity	*In vitro*: DPPH assay; IC_50_ 211–439 μg/ml; positive control: ascorbic acid, IC_50_ 38.65 μmol/L	Present good DPPH˙ scavenging activity, with IC_50_ values ranging from 211 to 439 μg/ml	Chen et al. (2020)
6	40% ethanol extract	*P. sinensis*	Stem	40% ethanol reflux extraction	HPLC, 5-*O*-caffeoylquinic acid 6.76 mg/g, scopolin 16.97 mg/g, chlorogenic acid 21.53 mg/g, 4-*O*-caffeoylquinic acid 7.84 mg/g, scopoletin 4.92 mg/g, 3,5-dicaffeoylquinic acid 12.41 mg/g, 3,4-dicaffeoylquinic acid 14.94 mg/g, 4,5-dicaffeoylquinic acid 18.17 mg/g	Anti-gout Activity	*In vivo*: monosodium urate crystal induced gout arthritis; ig, 1.0, 0.5, and 0.25 g/kg; positive control: colchicine 1.5 mg/kg	Regulate the release of inflammatory factors and oxygen free radicals to prevent and treat gouty arthritis by mediating the TLR2-MyD88 signaling pathway	Du et al. (2020)
7	80% methanol extract of ten samples	*P. sinensis*	Stem	80% methanol ultrasonic extraction	HPLC, chlorogenic acid, 4-*O*-caffeoylquinic acid, 5-*O*-caffeoylquinic acid, 3,4-dicaffeoylquinic acid, 4,5-dicaffeoylquinic acid, 3,5-dicaffeoylquinic acid, scopolin, scopoletin	Anti-gout Activity	*In vitro*: xanthine oxidase inhibitory activity assay; IC_50_ 26.7–45.5 mg/ml; positive control: allopurinol, IC_50_ 0.01 mmol/L	Present good xanthine oxidase inhibitory activity, with IC_50_ values ranging from 26.7 to 45.5 mg/ml	Chen et al. (2020)

#### 5.2.1 Anti-inflammatory and analgesic effects

In a previous study, our group adopted the xylene-induced mouse ear swelling model, the formalin-induced inflammation model, and the carrageenan-induced mice air pouch inflammation model to investigate the anti-inflammatory activity of 40% ethanol extracts of *P. sinensis* (extract **1**). We also applied the mouse acetic acid writhing model and the formalin-induced pain model to investigate its analgesic effects (Chen et al., 2013). We found that the oral administration of extract **1** (570 and 285 mg/kg) inhibits ear swelling in mice by 39.0% and 29.5%, respectively, and the induced inflammation in formalin mice by 37.3% and 30.8%, respectively. In the carrageenan-induced mice air pouch inflammation model, extract **1** significantly inhibits the synthesis of PGE2. Extract **1** significantly reduces the number of writings in mice and relieves phase II pain in the formalin-induced pain model. The 80% methanol ultrasonic extract of *P. sinensis* (extract **2**) inhibits LPS-induced RAW 264.7 release of NO at 25, 50, and 100 μg/ml, with inhibition of iNOS, COX-2, and IL-6 mRNA expression (Xue et al., 2017). However, this study lacked a positive control. COX-2 is a critical enzyme that catalyzes the conversion of arachidonic acid to prostaglandins, and this study confirmed the inhibitory effect of extract **1** on PGE2 synthesis. We reported that 40% ethanol extract of *P. sinensis* (extract **3**) inhibits the release of inflammatory mediators (NO, TNF-α, IL-1β, and IL-6) in LPS-induced RAW 264.7 cells (Hu et al., 2022). Extract **3** attenuates the severity, pathological changes, and release of cytokines (IL-6 and HIF-1α) during rheumatoid arthritis progression by regulating the PI3K/Akt and HIF-1 pathways (Hu et al., 2022).

There are many studies on the anti-inflammatory and analgesic efficacy of the extract of *P. sinensis in vitro* and *in vivo*. Compared with methotrexate, aspirin, and other positive control drugs, these extracts’ anti-inflammatory and analgesic effects are insignificant. Except for *P. sinensis*, species such as *P. spectabilis* have been recorded for the treatment of chest pain in folk medicine (Li et al., 1985); however, no experimental verification has been reported.

#### 5.2.2 Anti-oxidant activity

As a chronic inflammatory autoimmune disease, rheumatoid arthritis is closely related to oxidative stress (Peng et al., 2021). The 60% ethanol extract (extract **4**) of *P. paniculata* presented good anti-oxidant activity in DPPH assay, superoxide anion scavenging activity assay, nitric oxide scavenging activity assay, hydrogen peroxide scavenging assay and metal chelating activity (Kumar et al., 2015). In the superoxide anion scavenging assay, extract **4** exhibited more robust activity than the positive control butylated hydroxyanisole. In the hydrogen peroxide scavenging assay, extract **4** (IC_50_: 25.65 μg/ml) performed almost as well as gallic acid (IC_50_: 24.29 μg/ml). Our group also tested ten batches of 80% methanol extract (extract **5**) of *P. sinensis*, all of which showed good DPPH˙ scavenging activity, with IC_50_ values ranging from 211 to 439 μg/ml (Chen et al., 2020). However, the above-mentioned test method for anti-oxidant activity is based on chemical reaction *in vitro*, which is far from practical. Therefore, it is necessary to explore the antioxidant activity *in vivo* to clarify the molecular mechanisms of its antioxidant activity.

#### 5.2.3 Anti-gout effect

In a previous study, we applied the strategy of network analysis combined with experimental verification to study the mechanism of the 40% ethanol extract of *P. sinensis* (extract **6**) against gout. Extract **6** (0.25, 0.5, 1.0 g/g) dose-dependently reduced joint swelling in rats with monosodium urate (MSU) crystal-induced gout arthritis, with decreased serum MDA and IL-1β levels, and increased serum SOD, TGF-β, and IL-4 levels. By mediating the TLR2-MyD88 signaling pathway, it regulates the release of inflammatory factors and oxygen free radicals to prevent and treat gouty arthritis (Du et al., 2020). Because xanthine oxidase is a target for gout treatment, we tested the xanthine oxidase inhibitory activity of ten batches of the 80% methanol extract of *P. sinensis* (extract **7**), revealing its good activity, with IC_50_ values ranging from 26.7 to 45.5 mg/ml (Chen et al., 2020). The treatment of gout-related diseases is not traditionally applied to the genus *Porana*. Although the *in vitro* and *in vivo* experiments demonstrated the anti-gout potential of *P. sinensis*, it remains needs to be verified by clinical research. In addition, due to the different extraction methods of these extracts, the active components of anti-gout should be clarified in the future.

#### 5.2.4 Toxicity

Only acute toxicity of *P. sinensis* has been reported. No mice died with a single intragastric 40% ethanol extract of *P. sinensis* at 5 g/kg. The weights, behaviors, and anatomical examinations showed no apparent abnormalities within 14 days (Chen et al., 2013). However, because it is a medicinal plant, acute toxicity evaluation is insufficient, and chronic toxicity tests and clinical safety evaluations of *Porana* plants need to be performed.

In summary, the current research on the medicinal effects of *Porana* species concentrates on *P. sinensis*. Although *Porana* is widely distributed, its medicinal value is limited. Especially for *P. racemosa,* which enjoys abundant folk medicinal records and good development prospects, its systematic pharmacodynamic and clinical research is lacking. For the pharmacological study of the extract, to clarify its pharmacodynamic components, chemical analysis is required. Some studies have not provided quality control on the extracts, which would affect the reliability of these studies.

### 5.3 Pharmacological activities of the active constituents of *Porana* plants

To further analyze the pharmacological activities of this genus, we followed the pharmacological studies of compounds in this genus and discussed their correlation with the results of our network analysis. The results are summarized in Table 4.

**TABLE 4:** Bioactivities of the active compounds of *Porana* plants.

No.	Compounds	Activities	Dosage	Model	Positive control	Results	References
1	Scopoletin	Anti-inflammatory and anti-nociceptive activities	Ip: 1, 5, 10 mg/kg	Acetic acid induced writhing response, formalin test and λ-carrageenan induced paw edema in ICR mice	Indomethacin, ip, 10 mg/kg	Reduce the levels of NO, TNF-α, PGE2, and the protein expression of iNOS and COX-2 in the serum of carrageenan-induced paw edema mice, reduce the number of writhing in the mouse acetate writhing model, and the formalin-induced pain in the late phase	Chang et al. (2012)
2	Scopoletin	Anti-inflammatory activity	Ip: 0.1, 1, 5 mg/kg	Carrageenan-induced inflammation in the mouse model of pleurisy	Dexamethasone, ip, 0.5 mg/kg	Reduce serum NO, TNF-α and IL-1β levels, and inhibit p65, p38 phosphorylation in mouse lungs	Pereira dos Santos Nascimento et al. (2016)
3	Scopoletin	Anti-inflammatory activity	15, 30, 60 μmol/L	IL-1β induced fibroblast-like synoviocytes	-	Inhibit the production of IL-6, and the phosphorylation of p38, ERK, PKC and CREB	Dou et al. (2013)
4	Scopolin	Anti-inflammatory and anti-nociceptive activities	Ip: 25, 50, 100 mg/kg	Adjuvant-induced arthritis in rats	Dexamethasone, ip, 2 mg/kg	Alleviate the symptoms of adjuvant-induced arthritis by inhibiting the expression of IL-6, VEGF and FGF-2 in synovial tissue	Pan et al. (2009)
5	Umbelliferone	Anti-inflammatory activity	Oral administration: 20, 40 mg/kg	2,4-dinitrochlorobenzene and house dust mite extract treated mice	Dexamethasone, oral administration, 1 mg/kg	Reduce ear thickness, spleen size and weight, serum levels of IgE, IgG1, IgG2a, TNF-α, and IL-4, and mast cell infiltration	Lim et al. (2019)
6	Isofraxidin	Anti-inflammatory activity	1, 10, 50 μmol/L	IL-1β induced inflammatory response in human osteoarthritis chondrocytes	-	Block IL-1β-stimulated production of NO and PGE2, inhibit the expression of COX-2, iNOS, MMP-1, MMP-3, MMP-13, ADAMTS-4 and -5, suppress IκB-α degradation and NF-κB activation	Lin et al. (2018)
7	3,4-dicaffeoylquinic acid	Anti-inflammatory activity	35, 70, 140 μmol/L	LPS-induced RAW 264.7 cells		Inhibit NO/iNOS and PGE2/COX-2 pathways, block the nucleus translocation of NF-κB	Xue et al. (2019)
	3,5-dicaffeoylquinic acid						
	4,5-dicaffeoylquinic acid						
8	3,4-dicaffeoylquinic acid	Anti-inflammatory activity	Ig: 10, 20 mg/kg	Acute airway inflammation induced by ammonia liquor in mice	Prednisone acetate, ig, 10 mg/kg	Reduce the total leukocytes in the bronchoalveolar lavage fluid	Wu et al. (2016)
	3,5-dicaffeoylquinic acid						
	4,5-dicaffeoylquinic acid						
9	Eupatilin	Anti-inflammatory activity	1, 10, 100 μmol/L	LPS-stimulated macrophages	-	Inhibit the inflammatory modulators and NF-κB activation	Choi et al. (2011)
10	Eupatilin	Anti-inflammatory activity	1, 2, 5, 10 μmol/L	Murine arthritis model; human rheumatoid synoviocytes	-	Inhibit TNF-α-induced IL-6 and IL-1β mRNA expression, suppress osteoclast differentiation	Kim et al. (2015)
11	Quercetin	Anti-inflammatory activity	Oral gavage: 30 mg/kg	Collagen-induced arthritis in C57BL/6 mice	Methotrexate, ip, 0.5 mg/kg	Decrease serum TNF-a, IL-1β, IL-17, and MCP-1 levels	Haleagrahara et al. (2017)
12	Quercetin	Anti-inflammatory activity	Ip	Adjuvant-induced arthritis in C57BL/6 mice; mice air pouch model	Dexamethasone	Reduce neutrophil infiltration and promote the apoptosis of activated neutrophils by inhibiting neutrophil activities	Yuan et al. (2020)
13	β-ecdysterone	Anti-inflammatory activity	Subcutaneous injection: 0.6, 0.8, 1.0 mg/kg	Monoiodoacetate-induced osteoarthritis in rats	3-methyladenine, ip, 30 mg/kg; rapamycin, ip, 1 mg/kg	Inhibit 3-methyladenine-induced apoptosis of chondrocytes, down-regulate PI3K, p-AKT1, p-mTOR, p-p70S6K and caspase-3 expression, activate autophagy in chondrocytes	Tang et al. (2020)
14	*N-trans*-feruloyltyramine	Anti-inflammatory activity	6.25, 12.5, 25, 50 μg/ml	LPS-induced RAW 264.7 cells	-	Suppress mRNA expression of COX-2 and iNOS *via* suppression of AP-1 and JNK signaling pathway	Jiang et al. (2015)
15	Scopoletin	Anti-gout activity	Ip: 50, 100, 200 mg/kg; 30, 100, 300 μmol/L	Monosodium urate (MSU) crystal-induced inflammation in mouse air pouch model; MSU crystal-stimulated RAW 264.7 cells	Prednisolone, ip, 10 mg/kg	Decrease the number of neutrophils and mononuclear phagocytes of monosodium urate (MSU) crystal-induced inflammation in mouse; suppress the secretions of IL-1β, TNF-α, IL-6, PGE2 and NO in MSU crystal-stimulated RAW 264.7 cells, involving the suppression of NF-κB activation and blockade of MAPK signal pathway	Yao et al. (2012)
16	Scopoletin	Anti-gout activity	Ig: 4.9 mg/kg	Monosodium urate crystal induced gout arthritis in rats	Colchicine, ig, 1.5 mg/kg	Inhibit the production of serum MDA, IL-1β, TGF-1β, promote the release of SOD and IL-4, as well as inhibit the expression of TLR2 and MyD88 mRNA in rat joint synovium	Du et al. (2020)
17	3,5-dicaffeoylquinic acid	Anti-gout activity	60, 120, 240, 480, 960 μmol/L	Xanthine oxidase	Allopurinol	Exhibit weak xanthine oxidase inhibitory activity	Chen et al. (2014)
	3,4-dicaffeoylquinic acid						
	4,5-dicaffeoylquinic acid						
18	Scopoletin	Anti-cancer activity	3.56, 6.12, 12.5, 25, 50, 100 μmol/L	The normal cell line HCvEpC and the cervical cancer cell lines DoTc2, SiHa, HeLa, and C33A	-	Inhibit the growth of DoTc2, SiHa, HeLa, and C33A cells; the apoptotic cell death in HeLa cells has involved the up-regulation of Bax, caspase 3, 8, and 9, the downregulation of Bcl-2, and the blockade of the PI3K/AKT pathway	Tian et al. (2019)
19	Umbelliferone	Anti-cancer activity	5, 25, 50, 100, 150 μmol/L	Human renal carcinoma cells	-	Reduce cell proliferation and induce apoptotic events by regulating Ki67, MCM2, Bcl-2, CDK2, CyclinE1, CDK4, and CyclinD1	Wang et al. (2019)
20	Isofraxidin	Anti-cancer activity	5, 10, 20, 40, 80 μmol/L	Human colorectal cancer cells HT-29 and SW-480	-	Bate cell proliferation, induce cell apoptosis, and decrease the expression of anti-apoptotic protein Bcl-2; block Akt pathway *via* inhibition expression of *p*-Akt	Shen et al. (2017)
21	5-O-caffeoylquinic acid	Anti-cancer activity	1, 10, 50 μmol/L	p53 wild-type A549 and p53-deficient H1299 non-small cell lung cancer cells	-	Abrogate mitogen-stimulated invasion but not proliferation by the inactivation of p70^S6K^-dependent signaling pathway	In et al. (2016)
22	Chlorogenic acid	Anti-cancer activity	50, 100, 200 μmol/L	U2OS, Saos-2, and MG-63 osteosarcoma cells	-	Inhibit cell proliferation	Sapio et al. (2020)
23	Chlorogenic acid	Anti-cancer activity	40 mg/kg	4T1 breast cancer tumors in BALB/c mice	-	Participated in the induction of apoptosis, involving the increase of Bax/Bcl-2 ratio, the genes of p53 and caspase-3	Changizi et al. (2021)
24	Chlorogenic acid	Anti-cancer activity	250, 1000 μmol/L	HCT116 and HT29 human colon cancer cell lines	-	Inhibit the viability associated with the induction of cell cycle arrest at the S phase and the suppression of extracellular signal related kinase activation	Hou et al. (2017)
25	Eupatilin	Anti-cancer activity	40, 80, 120, 160, 200, 240, 280, 320 μmol/L	Human malignant glioma cell lines U251MG, U118, T98G, and U87MG	-	Inhibit the viability and proliferation of glioma cells by arresting the cell cycle at the G1/S phase, and disrupt the structure of the cytoskeleton and affect F-actin depolymerization *via* the p-LIMK/cofilin pathway	Fei et al. (2019b)
26	Eupatilin	Anti-cancer activity	12.5, 25, 50 μmol/L	Human prostate PC3, LNCaP cancer cells and prostatic epithelial RWPE-1 cells	-	Inhibit the proliferation, metastasis and spread of prostate cancer cells through modulation of PTEN and NF-κB pathway	Serttas et al. (2021)
27	Eupatilin	Anti-cancer activity	2.5, 5, 10, 20, 40 μmol/L; 10, 50 mg/kg	Human esophageal cancer cell line TE1; TE1 xenograft mouse model	-	Inhibit the Akt and ERK pathways	Wang et al. (2018b)
28	4ʹ-Hydroxywogonin	Anti-cancer activity	0.1, 1, 10 μg/ml	SW620 colorectal cancer cell	Wortmannin, 10 μmol/L	Reduce the viability, suppress the proliferation by disrupting PI3K/AKT pathway	Sun et al. (2018)
29	*N*-trans-feruloyltyramine	Anti-cancer activity	64, 128, 192, 256, 320 μmol/L	HepG2 and L02 human hepatoma cells	Taxol	Inhibit the proliferation	Gao et al. (2019)
30	Scopoletin	Anti-diabetic activity	Ig: 0.01 g/100 g diet	Streptozotocin induced diabetic mice	Metformin, 0.5 g/100 g diet	Reduce blood glucose and glycated hemoglobin, serum ALT, TNF-α, IL-6 levels, glucose intolerance, and hepatic lipid accumulation, down-regulate hepatic gene expression of triglyceride and cholesterol synthesis as well as inflammation (TLR4, MyD88, NF-κb1, TNF-α, and IL-6)	Choi et al. (2017)
31	Scopoletin	Anti-diabetic activity	Ig: 1 mg/kg	High fructose diet induce type 2 diabetes rats	-	Reduce blood glucose, insulin and lipid levels, involving the activation of IRS1, PI3K and AKT phosphorylation	Kalpana et al. (2019)
32	Scopoletin	Anti-diabetic activity	Ig: 10 mg/kg	Streptozotocin induced diabetes mice	Acarbose, Ig, 10 mg/kg	Inhibit the activity of α-glucosidase and α-amylase and reduce postprandial blood glucose levels	Jang et al. (2018)
33	Scopoletin	Phagocytic activity	50 μg/ml	Human U937 monocytic cell line	-	Enhance the phagocytic activity, which involving the down-regulation of seven genes (*CDC42, FCGR1A/FCGR1C, ITGA9, ITGB3, PLCE1, RHOD & RND3*) and up-regulation of five genes (*DIRAS3, ITGA1, PIK3CA, PIK3R3 & PLCD1*)	Alkorashy et al. (2020)
34	Scopoletin	Anti-fungal activity	12.5–200 μg/ml	*Candida tropicalis*	Fluconazole, 62.5–1000 μg/ml	Affect both planktonic and biofilm forms	Lemos et al. (2020)

#### 5.3.1 Anti-inflammatory and analgesic effects

The results of long-term folk medicinal and network analysis indicated that anti-inflammatory and analgesic effects are the primary medicinal effects of *Porana* plants. The intraperitoneal injection of scopoletin (compound **15**, 1, 5, 10 mg/kg) reduced serum levels of NO, TNF-α, and PGE2 of carrageenan-induced paw edema mice, and the protein expression of iNOS and COX-2 (Chang et al., 2012). Scopoletin reduced the amount of writhing in the mouse acetate writhing model and formalin-induced pain in the late phase. The anti-inflammatory and analgesic effects of scopoletin (10 mg/kg) are equivalent to that of indomethacin (10 mg/kg) (Chang et al., 2012). However, scopoletin was given by intraperitoneal injection, which would limit its application. In the carrageenan-induced mouse model of pleurisy, intraperitoneal injection of scopoletin (1 mg/kg) reduced serum NO, TNF-α, and IL-1β levels and inhibited p65, p38 phosphorylation in mouse lungs (Pereira dos Santos Nascimento et al., 2016). Dou et al. (2013) reported that scopoletin (15, 30, 60 μmol/L) significantly inhibited the production of IL-6 in fibroblast-like synoviocytes induced by IL-1β and the phosphorylation of p38, ERK, PKC, and CREB. These findings suggest that scopoletin might play a role by mediating the MAPK/PKC/CREB pathways. It should be noted that this study lacks a positive control. P38 MAPK is relevant to human inflammatory disease, and inhibition of p38 phosphorylation reduces gene expression of many inflammatory mediators (Dou et al., 2013). The regulatory effect of scopoletin on the MAPK signaling pathway is consistent with the results of network analysis. These findings suggest that scopoletin exerts anti-inflammatory and analgesic effects through multiple targets and pathways, indicating its good medicinal potential (Parama et al., 2022). However, due to the instability of scopoletin under physiological media and poor water solubility, its oral bioavailability is only 6.0%, severely restricting its medicinal application (Sakthivel et al., 2022). With the rapid development of pharmaceutical technology, new drug delivery systems have introduced possible applications of scopoletin in recent years. For example, there is a formulation of soluplus-based micelles for scopoletin, which increases its absorption, bioavailability, and tissue distribution 33-fold (Zeng et al., 2017). Pan et al. (2009) reported that intraperitoneal injection of scopolin (compound **16**, 50, and 100 mg/kg) alleviated the symptoms of adjuvant-induced arthritis in rats by inhibiting the expression of IL-6, VEGF, and FGF-2 in rat synovial tissue. Li et al. (2019) established an LC-MS/MS method for the simultaneous determination of scopolin and scopoletin in rat biomatrices, while the bioavailability of scopolin was exceptionally low.

There are also many reports on umbelliferone’s anti-inflammatory and analgesic activities (compound **17**) and isofraxidin (compound **20**). Oral administration of umbelliferone (20 and 40 mg/kg) for 28 days led to significant decreases in ear thickness, spleen size and weight, and serum levels of IgE, IgG1, IgG2a, TNF-α, and IL-4. There were also decreases in mast cell infiltration on 2,4-dinitrochlorobenzene and house dust mite extract-treated mice (Lim et al., 2019). Umbelliferone reduced the secretion of pro-inflammatory cytokines and chemokines in TNF-α/IFN-γ-treated HaCaT cells *via* the regulation of the MAPK, IkB-α/NF-κB, and STAT1 signaling pathways (Lim et al., 2019). There are many reports on isofraxidin in the treatment of osteoarthritis (Jin et al., 2018; Wang and Wang, 2021). For example, isofraxidin (1, 10, and 50 μmol/L) blocked IL-1β-stimulated production of NO and PGE2, inhibited the expression of COX-2, iNOS, MMP-1, MMP-3, MMP-13, ADAMTS-4 and -5, and suppressed IL-1β-induced IκB-α degradation and NF-κB activation in human osteoarthritis chondrocytes (Lin et al., 2018); it should be noted that there was no positive control group in this study. Pharmacokinetic studies demonstrated *in vivo* its rapid absorption after oral applications (Majnooni et al., 2020).

The HPLC fingerprints of the *Porana* plants (Figure 3) showed that quinic acid derivatives frequently appear in different *Porana* species. The anti-inflammatory, analgesic-related pharmacodynamics of chlorogenic acid has been reported in many studies and associated with the NF-κB, MAPK, and JNK/AP-1 signaling pathways; they have also been associated with the downregulation of TNF-α, COX-2, and PGE2 (Bagdas et al., 2020). Xue et al. (2019) applied the method of D101 macroporous resin to track the anti-inflammatory components in *P. sinensis*; Compounds **31–33** inhibited NO/iNOS and PGE2/COX-2 pathways, and the nuclear translocation of NF-κB was also blocked. Wu et al. (2016) reported that compounds **31–33** reduce mouse ammonia liquor-induced acute airway inflammation by reducing the total leukocytes in bronchoalveolar lavage fluid. Among these three compounds, 4,5-dicaffeoylquinic acid exhibited the most potent effect, suggesting that the structure-activity relationship requires further elaboration.

Seven flavonoids have been isolated from *Porana* species. Eupatilin (compound **23**) and quercetin (compound **25**) present diverse anti-inflammatory activities. Eupatilin exerts anti-inflammatory effects by regulating NF-κB (Choi et al., 2011), TLR4/MyD88 (Fei et al., 2019a), AMPK (Zhou et al., 2018), and by suppressing osteoclast differentiation (Kim et al., 2015), inhibiting oxidative stress (Ali et al., 2017). Although eupatilin has broad bioactivity, its oral bioavailability is only 2.7% (Wang et al., 2018a). Quercetin is a broad-spectrum anti-inflammatory and analgesic substance without specificity. Considering the folk medicinal application of *Porana* plants, we only focused on its application in arthritis. Quercetin decreased serum TNF-a, IL-1β, IL-17, and MCP-1 levels in a collagen-induced mouse arthritis model (Haleagrahara et al., 2017). The authors claimed that quercetin produces better activity than methotrexate, which might not be accurate due to the different doses and routes of administration (quercetin, Po with 30 mg/kg; methotrexate, Ip with 0.5 mg/kg). MCP-1 (chemokine ligand 2) has a critical role in inflammation (Singh et al., 2021). These studies confirmed the regulatory effect of *Porana* plants on the chemokine pathway in network analysis. Yuan et al. (2020) found that quercetin reduces neutrophil infiltration and promotes apoptosis in activated neutrophils; however, this study did not provide the dosage of quercetin and the positive control dexamethasone.

β-Ecdysterone (compound **1**) inhibited 3-methyladenine-induced apoptosis of chondrocytes, downregulated PI3K, *p*-AKT1, *p*-mTOR, *p*-p70S6K, and caspase-3 expression, and activated autophagy in chondrocytes in a rat model of monoiodoacetate-induced osteoarthritis (Tang et al., 2020). *N*-trans-feruloyltyramine (compound **35**) strongly suppressed mRNA expression of COX-2 and iNOS *via* suppression of AP-1 and the JNK signaling pathway in LPS-induced RAW 264.7 cells (Jiang et al., 2015).

There are several anti-inflammatory and analgesic active ingredients in *Porana* species, including coumarins, quinic acid derivatives, flavonoids, steroids, and amides. The results of the components (scopolin, umbelliferone, eupatilin, quercetin, *N*-trans-feruloyltyramine) pathways (PI3K-Akt, HIF-1, MAPK, chemokine) in our network analysis are consistent with the results of the literature review, which suggests the potential of *Porana* species in the treatment of arthritis. However, it should be noted that, although components such as scopoletin and eupatilin show good anti-inflammatory and analgesic effects, their bioavailability is relatively low. Further structural modification is needed, or new drug delivery systems should be developed to improve their bioavailability.

#### 5.3.2 Anti-gout effect

Intraperitoneal injection of scopoletin (compound **15**, 100, and 200 mg/kg) significantly lowered the number of neutrophils and mononuclear phagocytes of MSU-induced inflammation in a mouse air pouch model. The secretion of IL-1β, TNF-α, IL-6, PGE2, and NO were suppressed by scopoletin (30–300 μmol/L) at the transcriptional level in MSU-stimulated RAW 264.7 cells, mediated by the suppression of NF-κB activation and blockade of the MAPK signal pathway (Yao et al., 2012). In our previous study, we also found that scopoletin (4.9 mg/kg) inhibited the production of serum MDA, IL-1β, and TGF-1β, promoted the release of SOD and IL-4 and inhibited the expression of TLR2 and MyD88 mRNA in rat joint synovium (Du et al., 2020). In another study, we found that 3,4-dicaffeoylquinic acid (compound **31**, IC_50_: 0.32 mmol/L), 4,5-dicaffeoylquinic acid (compound **32**, IC_50_: 0.26 mmol/L), and 3,5-dicaffeoylquinic acid (compound **33**, IC_50_: 0.21 mmol/L) exhibited weak xanthine oxidase inhibitory activity (Positive control: allopurinol, IC_50_: 0.01 mmol/L) (Chen et al., 2014), partially explaining the phytochemistry of anti-gout activity.

In summary, scopoletin plays an anti-gout role primarily by regulating inflammatory pathways, and quinic acid derivatives have xanthine oxidase inhibitory activity. Due to a large amount of anti-inflammatory, analgesic, antioxidant, and xanthine oxidase-inhibiting ingredients, the genus *Porana* has excellent application prospects as anti-gout therapies. However, only *P. sinensis* has been reported to treat acute gouty arthritis. Therefore, the anti-gout efficacy of other species in this genus must be further explored.

#### 5.3.3 Anti-cancer activity

Scopoletin (compound **15**) inhibited the growth of cervical cancer cell lines, including DoTc2, SiHa, HeLa, and C33A cells, with the IC_50_ values ranging from 7.5 to 25 μmol/L. The apoptotic cell death in HeLa cells induced by scopoletin involved the upregulation of Bax, caspase 3, 8, and 9, the downregulation of Bcl-2, and the blockade of the PI3K/Akt pathway. Scopoletin also caused cell cycle arrest at the G2/M phase and inhibited cell migration (Tian et al., 2019). Umbelliferone (compound **17**) exerted anti-cancer effects on various cells and animal models through induction of apoptosis, cell cycle arrest, reduction of cell proliferation, and inhibition of the release of inflammatory factors. For example, treating human renal carcinoma cells with umbelliferone-induced dose-dependent decreases in Ki67, MCM2, Bcl-2, CDK2, CyclinE1, CDK4, and CyclinD1 and an increase in Bax (Wang et al., 2019). Isofraxidin (compound **20**, 5–80 μmol/L) significantly bate cell proliferation, induced cell apoptosis, and decreased the expression of anti-apoptotic protein Bcl-2 in human colorectal cancer cell lines (HT-29 and SW-480). Isofraxidin blocks the Akt pathway *via* inhibition expression of *p*-Akt (Shen et al., 2017).

There are many reports on the anti-cancer properties of quinic acid derivatives in *Porana* species. 5-*O*-caffeoylquinic acid (compound **30**) abrogated mitogen-stimulated invasion but not proliferation in p53 wild-type A549 and p53-deficient H1299 NSCLC cells. The anti-invasive activity of 5-*O*-caffeoylquinic acid in A549 cells might be mediated by the inactivation of the p70^S6K^-dependent signaling pathway (In et al., 2016). Chlorogenic acid (compound **28**) inhibited the proliferation of U2OS, Saos-2, and MG-63 osteosarcoma cells (50, 100, 200 μmol/L) (Sapio et al., 2020). This compound also participates in the apoptosis of 4T1 breast cancer tumors in BALB/c mice, involving the increase of the Bax/Bcl-2 ratio and the genes for p53 and caspase-3 (Changizi et al., 2021); it inhibits the viability of HCT116 and HT29 colon cancer cell lines associated with the induction of cell cycle arrest at the S phase and the suppression of extracellular signal-related kinase activation (Hou et al., 2017). These findings suggest that caffeoylquinic acids exhibit relatively broad anti-cancer activity, with targeted cancer types including lung cancer, osteosarcoma, breast cancer, and colon cancer. Chlorogenic acid inhibits cell proliferation and blocks the cell cycle; however, 5-*O*-caffeoylquinic acid does not inhibit cell proliferation. As isomers, the difference in antiproliferative effect between these two compounds deserves further explanation.

The flavonoid eupatilin (compound **23**) inhibits the viability and proliferation of glioma cells by arresting the cell cycle at the G1/S phase. Eupatilin disrupts the structure of the cytoskeleton and affects F-actin depolymerization *via* the *p*-LIMK/cofilin pathway (Fei et al., 2019b). However, this study did not report a proapoptotic effect of eupatilin on glioma, which was inconsistent with other studies. Eupatilin (12.5, 25, 50 μmol/L) inhibits the proliferation, metastasis, and spread of prostate cancer cells through modulation of PTEN and NF-κB signaling (Serttas et al., 2021); it blocks the proliferation of esophageal cancer TE1 cells associated with the inhibition of the Akt and ERK pathways (Wang et al., 2018b). Another flavonoid, 4ʹ-hydroxywogonin (compound **24**), reduced the viability and suppressed the proliferation of SW620 colorectal cancer cells angiogenesis by disrupting PI3K/Akt signaling, while the expression of VEGF-A decreased dose-dependently (Sun et al., 2018). Based on this study, it could be presumed that the anti-angiogenic activity of PI3K inhibitors was at least partially mediated by their capacity to reduce VEGF levels.

*N*-trans-feruloyltyramine (compound **35**) inhibits the proliferation of HepG2 cells with an IC_50_ of 194 ± 0.894 μmol/L, which was significantly lower than the positive control taxol (IC_50_: 26 ± 0.128 μmol/L) (Gao et al., 2019). Comparing the results on HepG2 and LO2 cells revealed that *N*-trans-feruloyltyramine might have selective cytotoxic effects.

In summary, there are many anti-cancer active components in *Porana* plants, including coumarins, quinic acid derivatives, and flavonoids. Of these, scopoletin, umbelliferone, chlorogenic acid, and eupatilin have many reports on their anti-cancer activity. These components are widely distributed in nature and are not specific. Since the related research mostly stays at the level of *in vitro* research, and more *in vivo* research and clinical studies are needed.

#### 5.3.4 Anti-diabetic activity

In the streptozotocin-induced diabetic mice model, scopoletin (compound **15**, 0.01 g/100 g diet) reduced blood glucose and glycated hemoglobin, glucose intolerance, hepatic lipid accumulation and downregulated hepatic gene expression of triglyceride and cholesterol synthesis and inflammation (TLR4, MyD88, NF-κb1, TNF-α, and IL-6). These results suggest that scopoletin protects against diabetes-induced steatosis and inflammation by inhibiting lipid biosynthesis and the TLR4-MyD88 pathway (Choi et al., 2017). However, this was a single-dose study with substantial differences in the dosage of the positive control metformin (0.5 g/100 g diet) and scopoletin, which cannot be used for comparison. In another study, scopoletin (1 mg/kg) reduced blood glucose, insulin, and lipid levels in high-fructose diet-induced type 2 diabetes, involving the activation of IRS1, PI3K, and Akt phosphorylation (Kalpana et al., 2019). Scopoletin inhibited the activity of α-glucosidase and α-amylase and reduced postprandial blood glucose levels in streptozotocin-induced diabetes mice. Unfortunately, the IC_50_ value of scopoletin was 85.12 and 37.36 μmol/L for α-glucosidase and α-amylase, which were lower than acarbose (Jang et al., 2018), indicating that its potential is limited. Another study reported that scopoletin stimulated insulin secretion *via* a K^+^ATP channel-dependent pathway in INS-1 pancreatic β cells (Park et al., 2022). Scopoletin could play a role in treating diabetes by stimulating insulin secretion, inhibiting α-glucosidase and α-amylase, and downregulating triglyceride and cholesterol synthesis and inflammation. However, the inhibitory effect of scopoletin on α-glucosidase and α-amylase would be weaker than that of the positive control drug acarbose.

There are many reports on the efficacy and mechanism of chlorogenic acid (compound **28**), quercetin (compound **25**), and rutin (compound **27**) in the treatment of diabetes. For example, quercetin stimulated insulin secretion (Kittl et al., 2016), alleviated ferroptosis in pancreatic cells (Li et al., 2020), and ameliorated diabetic encephalopathy through the SIRT1/ER stress pathway (Hu et al., 2020). Rutin decreased carbohydrate absorption from the small intestine, inhibited tissue gluconeogenesis, increased tissue glucose uptake, stimulated insulin secretion from beta cells, and protected pancreatic islets against degeneration (Ghorbani, 2017). Chlorogenic acid prevented diabetic nephropathy (Bao et al., 2018), rescued sensorineural auditory function, attenuated insulin resistance, and modulated glucose uptake (Hong et al., 2017).

In summary, many anti-diabetic ingredients are found in *Porana* plants, including coumarins, quinic acid derivatives, and flavonoids. The content of coumarins and quinic acid derivatives is relatively high in the genus *Porana*, suggesting that this genus could be used to treat diabetes. The network analysis shows that the pathways regulated by the chemical components of *Porana* plants play an essential role in diabetes treatment. For example, the PI3K/Akt pathway damaged in various body tissues leads to obesity and type 2 diabetes as the result of insulin resistance; in turn, insulin resistance exacerbates the PI3K/Akt pathway, forming a vicious circle (Huang et al., 2018). The progression of diabetes and its complications can be prevented or treated by modulating HIF-1 expression or activity (Catrina and Zheng, 2021). However, apart from the pharmacological or clinical studies of these compounds, there are no reports on the application of *Porana* plants in diabetes treatment; relevant research needs to be performed.

#### 5.3.5 Other activities

Alkorashy et al. (2020) used transcriptomic methods to study the effect of scopoletin (compound **15**) on the phagocytosis of stimulated U937-derived macrophages. Scopoletin enhanced the phagocytic activity, involving the downregulation of seven genes (*CDC42*, *FCGR1A/FCGR1C*, *ITGA9*, *ITGB3*, *PLCE1*, *RHOD,* and *RND3*) and upregulation of five genes (*DIRAS3*, *ITGA1*, *PIK3CA*, *PIK3R3,* and *PLCD1*). These results provide a basis for applying scopoletin in treating cancer progression and metastasis, autoimmune disorders, pelvic organ prolapse, and cystic fibrosis. *ITGB3* is upregulated in pelvic organ prolapse disorders in women, and the downregulation of these genes supports the folk medicinal application of *P. spectabilis* in the treatment of uterine prolapse. Scopoletin also acts as an anti-fungal phytocompound against a multidrug-resistant strain of *Candida tropicalis,* with properties affecting planktonic and biofilm forms of this pathogen (Lemos et al., 2020).

## 6 Conclusion

The genus *Porana* is abundant in natural resources and is widely distributed in Asia, Africa, Oceania, America, and other regions. In China and India, this genus has several medicinal records. Currently, only the chemical composition, efficacy, and quality control of *P. sinensis* have been systematically reported, while the medicinal value of other species in this genus has not yet been explored. Therefore, we systematically reviewed this genus’s traditional and current use, chemical compositions, and pharmacological activities. We applied network analysis to predict the key targets and pathways of chemical components in this genus to clarify the research status of *Porana* species and highlight the directions for the rational medicinal development of this genus.

Regarding chemical components, only five species of genus *Porana* have been reported, with 59 compounds isolated and identified, including steroids, coumarins, flavonoids, quinic acid derivatives, and amides. Combined with the fingerprints (Figure 3), coumarins and quinic acid derivatives are widely distributed in this genus, while steroids have only been reported in *P. discifera*. Because the research on chemical constituents is the forerunner of medicinal value development, the phytochemical study of other species in this genus needs to be performed.

In terms of pharmacological effects, the extracts of *Porana* plants exhibit anti-inflammatory, analgesic, antioxidant, and anti-gout activities. However, studies on the pharmacological effects of *Porana* plants are focused on *P. sinensis*, and there are few pharmacological studies on other species. Especially for plants with extensive folk medicinal records (such as *P. racemosa*), detailed pharmacodynamic research needs to be performed. The chemical constituents of *Porana* present anti-inflammatory, analgesic, anti-gout, anti-cancer, and diabetes treatment activities. Gout and diabetes treatment are not the traditional medicinal applications of *Porana* plants. However, this genus contains chemical substances with appropriate biological activities. Therefore, we speculate that this genus has the potential to develop in the direction of anti-gout and anti-diabetes. Future research needs to investigate different species’ anti-gout and anti-diabetic efficacy, explain their mechanism of action, and systematically elucidate their active components.

Network analysis showed that steroids, flavonoids, amides, coumarins, and other components maybe be relevant for anti-inflammatory, analgesic, anti-gout, anti-cancer, and diabetes treatment activities of *Porana* plants. Their targets include GSK3B, EGFR, MAPK1, IL2, HSPA8, MMP9, HK1, GAPDH, TNF, ADORA3, and their pathways include PI3K-Akt, HIF-1, estrogen, and MAPK. The enriched targets and pathways are consistent with the results of our literature review.

In summary, *Porana* plants are abundant in natural resources and are widely recorded in folk medicine; nevertheless, the study of their medicinal value is limited. Research on the systematic chemical constituents of this genus is urgently needed. Anti-inflammatory, analgesic, anti-gout, anti-cancer, and diabetes treatments are critical directions for future study.

## Abbreviations

Akt, protein kinase B; COX-2, cyclooxygenase-2; C-T-P, compound-target-pathway; FGF-2, fibroblast growth factor 2; HIF, hypoxia inducible factor; HPLC, high performance liquid chromatography; Ig, intragastric administration; IL-6, interleukin 6; iNOS, inducible nitric oxide synthase; Ip, intraperitoneal injection; KEGG, Kyoto Encyclopedia of Genes and Genomes; LPS, lipopolysaccharide; MAPK, mitogen-activated protein kinases; MDA, malonic dialdehyde; MSU, monosodium urate; MyD88, myeloid differentiation factor 88; NF-κB, nuclear transcription factor-κB; NO, nitric oxide; PGE2, prostaglandin E2; PI3K, phosphoinositide 3-kinase; PPI, protein-protein interaction; SOD, superoxide dismutase; STAT, signal transducer and activator of transcription; TLR2, toll-like receptor 2; TNF-α, tumor necrosis factor-α; VEGF, vascular endothelial growth factor.
