# Supplementary material for: The genus Porana (Convolvulaceae) - A phytochemical and pharmacological review
Source: Front Pharmacol. 2022 Oct 18;13:998965. doi: 10.3389/fphar.2022.998965 (PMC9622789; doi:10.3389/fphar.2022.998965)
Supplement: Supplementary file 1 [file DataSheet1.DOCX]

**Table S1 The information of top 20 significant KEGG enrichment analysis (David, *P*-value＜0.01)**

| **ID** | **Description** | **GeneRatio** | ***P*-value** | **Count** | **Genes** |
| --- | --- | --- | --- | --- | --- |
| hsa04151 | PI3K-Akt | 0.24 | 7.95E-14 | 32 | GSK3B, HSP90AB1, ITGA2B, PIK3R1, FGF2, RELA, EGFR, PPP2CA, NRAS, CCND1, KDR, AKT1, MAPK1, JAK2, MAPK3, MAP2K1, HSP90AA1, NOS3, PRKCA, IL2, MTOR, NFKB1, VEGFA, IL6, PIK3CA, CDK4, KIT, CDK2, MDM2, GRB2, TLR4, BCL2L1 |
| hsa04915 | Estrogen | 0.17 | 2.24E-18 | 23 | HSPA8, MAP2K1, JUN, HSP90AA1, HSP90AB1, NOS3, SRC, MMP2, PRKCD, PIK3R1, OPRM1, ESR1, MMP9, EGFR, ESR2, NRAS, PIK3CA, AKT1, MAPK1, GRB2, CALM1, PRKACA, MAPK3 |
| hsa04066 | HIF-1 | 0.16 | 2.00E-17 | 22 | MAP2K1, CREBBP, PRKCB, NOS3, STAT3, PRKCA, PIK3R1, HIF1A, EGFR, MTOR, RELA, NFKB1, HK1, VEGFA, IL6, PIK3CA, ERBB2, AKT1, MAPK1, GAPDH, TLR4, MAPK3 |
| hsa04014 | Ras | 0.16 | 7.65E-10 | 22 | MAP2K1, PRKCB, PRKCA, PIK3R1, FGF2, EGFR, RELA, NFKB1, VEGFA, NRAS, MAPK8, PIK3CA, KIT, KDR, ABL1, AKT1, MAPK1, GRB2, CALM1, PRKACA, BCL2L1, MAPK3 |
| hsa04068 | FoxO | 0.16 | 3.03E-13 | 21 | MAP2K1, CREBBP, PLK1, STAT3, PIK3R1, MAPK14, SIRT1, FOXO1, EGFR, IL6, CCNB1, NRAS, MAPK8, PIK3CA, CCND1, CDK2, MDM2, AKT1, MAPK1, GRB2, MAPK3 |
| hsa04010 | MAPK | 0.16 | 3.31E-08 | 21 | HSPA8, NTRK2, MAP2K1, JUN, PRKCB, PRKCA, MAPK14, FGF2, TNF, EGFR, RELA, NFKB1, NRAS, MAPK8, CASP3, AKT1, MAPK1, GRB2, MAPT, PRKACA, MAPK3 |
| hsa04919 | Thyroid hormone | 0.15 | 1.87E-13 | 20 | GSK3B, MAP2K1, CREBBP, PRKCB, HDAC1, SRC, PRKCA, PIK3R1, HIF1A, ESR1, FOXO1, MTOR, NRAS, PIK3CA, CCND1, MDM2, AKT1, MAPK1, PRKACA, MAPK3 |
| hsa04062 | Chemokine | 0.15 | 1.08E-09 | 20 | LYN, CCR1, GSK3B, MAP2K1, SRC, PRKCD, STAT3, PIK3R1, RELA, NFKB1, NRAS, PIK3CA, CXCR1, CXCR2, AKT1, MAPK1, GRB2, JAK2, PRKACA, MAPK3 |
| hsa04015 | Rap1 | 0.15 | 8.39E-09 | 20 | MAP2K1, PRKCB, SRC, ITGA2B, PRKCA, PIK3R1, MAPK14, FGF2, EGFR, VEGFA, NRAS, PIK3CA, CNR1, KIT, KDR, AKT1, MAPK1, CALM1, DRD2, MAPK3 |
| hsa04917 | Prolactin | 0.14 | 2.74E-16 | 19 | GSK3B, MAP2K1, SRC, STAT3, PIK3R1, MAPK14, ESR1, RELA, ESR2, NFKB1, NRAS, MAPK8, PIK3CA, CCND1, AKT1, MAPK1, GRB2, JAK2, MAPK3 |
| hsa04660 | T cell receptor | 0.14 | 1.82E-13 | 19 | GSK3B, MAP2K1, JUN, PIK3R1, MAPK14, TNF, IL2, RELA, NFKB1, NRAS, PTPRC, PIK3CA, CDK4, LCK, AKT1, MAPK1, GRB2, FYN, MAPK3 |
| hsa04668 | TNF | 0.14 | 6.17E-13 | 19 | MAP2K1, JUN, PIK3R1, PTGS2, MAPK14, SELE, TNF, MMP9, RELA, NFKB1, MMP14, IL6, MAPK8, CASP8, PIK3CA, CASP3, AKT1, MAPK1, MAPK3 |
| hsa04722 | Neurotrophin | 0.14 | 4.72E-12 | 19 | NTRK2, GSK3B, MAP2K1, JUN, PRKCD, PIK3R1, PSEN1, MAPK14, RELA, NFKB1, NRAS, MAPK8, PIK3CA, ABL1, AKT1, MAPK1, GRB2, CALM1, MAPK3 |
| hsa04012 | ErbB | 0.13 | 2.16E-13 | 18 | GSK3B, MAP2K1, JUN, PRKCB, SRC, PRKCA, PIK3R1, EGFR, MTOR, NRAS, MAPK8, PIK3CA, ERBB2, ABL1, AKT1, MAPK1, GRB2, MAPK3 |
| hsa04071 | Sphingolipid | 0.13 | 4.98E-11 | 18 | MAP2K1, PRKCB, NOS3, PRKCA, PIK3R1, MAPK14, TNF, RELA, NFKB1, PPP2CA, NRAS, MAPK8, PIK3CA, ADORA3, AKT1, MAPK1, FYN, MAPK3 |
| hsa04912 | GnRH | 0.13 | 6.43E-12 | 17 | MAP2K1, JUN, PRKCB, SRC, MMP2, PRKCD, PRKCA, MAPK14, EGFR, MMP14, NRAS, MAPK8, MAPK1, GRB2, CALM1, PRKACA, MAPK3 |
| hsa04370 | VEGF | 0.11 | 3.12E-12 | 15 | MAP2K1, PRKCB, NOS3, SRC, PRKCA, PIK3R1, PTGS2, MAPK14, VEGFA, NRAS, PIK3CA, KDR, AKT1, MAPK1, MAPK3 |
| hsa04664 | Fc epsilon RI | 0.11 | 1.53E-11 | 15 | LYN, MAP2K1, PRKCB, PRKCA, PIK3R1, MAPK14, TNF, NRAS, MAPK8, PIK3CA, AKT1, MAPK1, GRB2, FYN, MAPK3 |
| hsa04620 | Toll-like receptor | 0.11 | 7.23E-09 | 15 | MAP2K1, JUN, PIK3R1, MAPK14, TNF, RELA, NFKB1, IL6, MAPK8, CASP8, PIK3CA, AKT1, MAPK1, TLR4, MAPK3 |
| hsa04662 | B cell receptor | 0.10 | 3.67E-09 | 13 | LYN, GSK3B, MAP2K1, JUN, PIK3R1, RELA, NFKB1, NRAS, PIK3CA, AKT1, MAPK1, GRB2, MAPK3 |

**Table S2 The topological parameter analysis of C-T-P for *Porana* plants**

| **Number** | **Node** | **Betweenness centrality**  **(BC)** | **Closeness centrality**  **(CC)** | **Degree centrality**  **(DC)** |
| --- | --- | --- | --- | --- |
| 1 | 2-deoxy-20-hydroxyecdysone | 0.0265 | 0.4072 | 18 |
| 2 | β-ecdysterone-25-acetate | 0.0256 | 0.4188 | 17 |
| 3 | *N-trans*-feruloyltyramine | 0.0362 | 0.4118 | 15 |
| 4 | Eupatilin | 0.0266 | 0.4164 | 15 |
| 5 | Ethyl 4'-hydroxy-3'-methoxycinnamate | 0.0234 | 0.4050 | 15 |
| 6 | 4ʹ-Hydroxywogonin | 0.0231 | 0.4188 | 15 |
| 7 | *N-trans*-coumaroyltyramine | 0.0215 | 0.3984 | 15 |
| 8 | β-ecdysterone-2-acetate | 0.0116 | 0.3941 | 14 |
| 9 | Quercetin | 0.0140 | 0.3984 | 13 |
| 10 | (*E*)-*N*-2-(2,3-dihydroxyphenyl) ethyl cinnamamide | 0.0129 | 0.3962 | 13 |
| 11 | Ethyl caffeate | 0.0134 | 0.3984 | 12 |
| 12 | β-ecdysterone | 0.0113 | 0.3941 | 12 |
| 13 | β-ecdysterone-3-acetate | 0.0085 | 0.3899 | 12 |
| 14 | 2,3-acetonide-β-ecdysterone | 0.0082 | 0.3703 | 12 |
| 15 | Umbelliferone | 0.0100 | 0.3879 | 11 |
| 16 | Scopolin | 0.0143 | 0.3798 | 10 |
| 17 | 4-methoxycinnamic acid | 0.0088 | 0.3838 | 8 |
| 18 | MAPK1 | 0.0562 | 0.4788 | 32 |
| 19 | PIK3CA | 0.0395 | 0.4637 | 28 |
| 20 | AKT1 | 0.0306 | 0.4608 | 26 |
| 21 | MAP2K1 | 0.0306 | 0.4608 | 25 |
| 22 | MAPK3 | 0.0290 | 0.4551 | 24 |
| 23 | EGFR | 0.0408 | 0.4468 | 24 |
| 24 | MMP2 | 0.0638 | 0.4212 | 22 |
| 25 | PRKCA | 0.0473 | 0.4261 | 21 |
| 26 | ESR2 | 0.0411 | 0.4095 | 21 |
| 27 | GSK3B | 0.0255 | 0.4336 | 20 |
| 28 | MAPK14 | 0.0197 | 0.4336 | 20 |
| 29 | ESR1 | 0.0398 | 0.4141 | 20 |
| 30 | PIK3R1 | 0.0164 | 0.4388 | 19 |
| 31 | NRAS | 0.0125 | 0.4261 | 18 |
| 32 | SRC | 0.0155 | 0.4141 | 17 |
| 33 | PTGS2 | 0.0369 | 0.3962 | 17 |
| 34 | MMP9 | 0.0196 | 0.4095 | 16 |
| 35 | TNF | 0.0313 | 0.4027 | 16 |
| 36 | KDR | 0.0232 | 0.4005 | 15 |
| 37 | ADORA3 | 0.0242 | 0.3899 | 12 |
| 38 | PI3K-Akt signaling pathway | 0.0661 | 0.4495 | 28 |
| 39 | HIF-1 signaling pathway | 0.0490 | 0.4236 | 22 |
| 40 | Estrogen signaling pathway | 0.0371 | 0.4286 | 21 |
| 41 | MAPK signaling pathway | 0.0329 | 0.4212 | 21 |
| 42 | Chemokine signaling pathway | 0.0376 | 0.4164 | 20 |
| 43 | Thyroid hormone signaling pathway | 0.0293 | 0.4072 | 19 |
